# Multidrug-Resistant *Vibrio* spp. carrying carbapenemase genes in aquacultured shrimp and their zoonotic importance

**DOI:** 10.1038/s41598-026-62098-x

**Published:** 2026-07-22

**Authors:** Maha M. Rezk, Dalia Hamza, Elshaimaa Ismael, Nehal H. Harfoush, Fatma A. El Gohary

**Affiliations:** 1 Department of Fish Diseases, Animal Health Research Institute AHRI, Agriculture Research Centre ARC, Damietta, 12619 Egypt; 2 Department of Zoonoses, Faculty of Veterinary Medicine, Cairo University, Giza, 12211 Egypt; 3 Department of Veterinary Hygiene and Management, Faculty of Veterinary Medicine, Cairo University, Giza, 12211 Egypt; 4 Department of Microbiology and immunology, Faculty of Medicine, Damietta University, Damietta, 34517 Egypt; 5 Department of Hygiene and Zoonoses, Faculty of Veterinary Medicine, Mansoura University, Mansoura, 35516 Egypt

**Keywords:** *Vibrio* spp*.*, Aquacultured shrimp, Multidrug resistance (MDR), Carbapenemase genes, One Health, Microbiology, Molecular biology

## Abstract

In the present study, samples (hepatopancreas and muscles) from 100 cultured shrimp (*Penaeus indicus*) were collected. The water samples (n = 30) were obtained from the same shrimp aquaculture farms located at Damietta Governorate in Egypt. An additional 30 samples of human stool were also taken from fish workers who consumed or handled shrimp in the farms. Traditional and regular molecular methods were used for the isolation of *Vibrio* spp. The investigation identified 165 (63.5%) *Vibrio* spp. with predominance of *V. fluvialis* (41.2%), *V. alginolyticus* (31.5%) and *V. parahaemolyticus* (7.9%). Findings of large-scale multidrug resistance (77.5% of isolates) and also of the presence of carbapenemase genes (*bla*_*KPC*_, *bla*_*NDM*_) and extended-spectrum beta-lactamase genes (*bla*_*CTX-M*_, *bla*_*TEM*_) were important discoveries. This strong resistance, due to the fact that a lot of pressure has been exerted on the aquaculture through the use of antibiotics, demonstrates the ineffectiveness of antibiotics in combating vibriosis in shrimp farming. The clinical signs observed in shrimps were red and fragile shells, and pale hepatopancreas. Histopathological analyses corroborated the presence of sloughed hepatopancreas epithelium, vacuolation, oedema and hemocytic infiltration. Regarding sequence similarity, the *gyrB* gene was sequenced, and all of the shrimp hepatopancreas isolates showed 100% sequence identity with the human stool isolates, which indicates potential genetic relatedness of *V. fluvialis* isolates. However, because of the conserved nature of *gyrB*, higher resolution tools are needed to verify transmission (e.g., MLST or WGS). The results of this study show that ecological management strategies and improved farm hygiene practices can be more effective intervention strategies than antibiotic use, providing valuable insights for sustainable shrimp aquaculture and public health interventions.

**Supplementary Information:**

The online version contains supplementary material available at 10.1038/s41598-026-62098-x.

## Introduction

Egypt’s brackish water aquaculture contributes significantly to Mediterranean aquaculture production which is accounted for 16.6% of total output^1^. Shrimp is a highly consumed seafood around the world and intensive farming has boosted demand, potentially making them more susceptible to disease. Shrimp farming is an emerging industry in Egypt, which has an extensive Mediterranean coastline of ~1050 km. In Egypt, about 80% of the production is in semi-intensive aquaculture systems. Although high production of shrimp, the infection by bacteria can be a problem and can cause significant economic losses^2^.

*Vibrio* species are ubiquitous Gram-negative halophilic bacteria found in coastal and estuarine water and have been proposed as "microbial barometer of climate change"^3^. As global temperatures are expected to increase by 1.5ºC in the coming years^4^, the distribution and abundance of pathogenic *Vibrio* spp. are predicted to spread and grow, which will increase the risks of infectious disease outbreaks in aquaculture and in humans, due to climate change^5^^,^^6^.

*Vibrio* bacteria are salt loving with curved shape microbes that inhabit water and are found in seafood like fish, shellfish, and crustaceans^7^. Vibrios are free-living or symbiotic in aquatic environments but can rapidly disseminate in intensive culture settings through water and direct host contact, affecting multiple marine organisms and posing significant risks to aquaculture biosecurity^8^^,^^9^. It causes severe economic losses in shrimp industry because of shrimp mortality and treatment cost.

*Penaeus indicus* (Indian prawn) is affected by bacterial and viral pathogens^10^. *Vibrio* spp. are major bacterial pathogens in shrimp aquaculture, particularly causing Acute Hepatopancreatic Necrosis Disease (AHPND), a condition that was originally described as Early Mortality Syndrome (EMS), often triggered by stressors such as environmental fluctuations and compromised host immunity^11,12^. Shrimp hepatopancreas is considered a vital organ in shrimp metabolism, immunity and antioxidant process. Antioxidant enzymes (Catalase (CAT) and superoxide dismutase (SOD) scavenge harmful free radicals^13,14^. During 2021, *Vibrio* spp. was isolated from shrimp hepatopancreas reared in commercial farms with high shrimp mortality in Damietta Governorate^15^. Bacteriological examination demonstrated that 56.6% of the isolates were *V. parahaemolyticus*, while 43.3% were *V. alginolyticus*.

Eating raw or undercooked shellfish, shrimp, and other seafood contaminated with harmful bacteria has been linked to many cases of food poisoning in humans^16^. Shrimp can become contaminated if they’re grown, processed or handled in unclean or unsafe conditions^8^. This contamination includes *Vibrio* bacteria, which not only may spoil food but can also spread serious illnesses like cholera and other infections^17^.

Out of the 30 known *Vibrio* species, at least 12 can make people sick, with the isolates; *V. parahaemolyticus*, *V. cholerae*, and *V. vulnificus* being the most dangerous^18,19^ causing severe illnesses, like Cholera (a life-threatening diarrheal disease caused by *V. cholerae* as mentioned^20^, gastroenteritis (food poisoning with vomiting, diarrhea, and fever from *V. parahaemolyticus* described by Tan et al.^21^. Moreover, flesh-eating and deadly bloodstream infections (from *V. vulnificus*, especially in people who eat raw seafood as reported by Ralph and Currie^22^ and wound infections (when open cuts are exposed to seawater contaminated with *V. alginolyticus*, reported by Vu et al.^23^ were documented. Because these bacteria are naturally present in marine environments, proper handling, cooking, and storage of seafood are crucial to prevent infections.

Beyond their role as causative agents of human infections, *Vibrio* species are also a major concern in aquaculture systems. The extensive use of antibiotics in shrimp cultivation has resulted in the emergence of multidrug resistant (MDR) Vibrios, making treatment difficult and causing food safety concerns. The most common antimicrobials are quinolones, tetracyclines and chloramphenicol. Unresponsible use promotes the spread of resistance genes, requiring responsible antimicrobial stewardship^24^.

The carbapenems are one of the most potent antibiotics available and are usually reserved as a last resort for patients with serious infections from drug resistant bacteria, including those that produce extended-spectrum beta-lactamases (ESBLs). The *TEM*, *SHV*, *CTX-M* or *OXA* genes code for β-lactamases which provide resistance by cleaving the β-lactam antibiotics^25^.

The growing prevalence of carbapenem-resistant *Vibrio* is now a significant global health concern; this resistance is mainly due to carbapenems-special enzymes produced by certain bacteria to break down and neutralize these antibiotics^26^. The most alarming carbapenems are *NDM-1*(most prevalent in *Vibrio* spp.), *OXA*, *VIM*, *VCC*, *IMP*, *GES*, *VMB*, *VAM* and *KPC*^27^. *Vibrio* spp. were reported as having ESBL genes such as *CTX*^28^.

Molecular diagnostic tools, especially PCR and sequence-based methods, offer precise and rapid identification of *Vibrio* species, and support epidemiological investigations and management strategies^29^. Therefore, the present study provides critical insights into AMR dynamics and one health challenges in aquaculture systems through (1) investigating the prevalence and diversity of *Vibrio* spp. in shrimp (*P. indicus*), aquaculture water, and human fish workers clinical samples in Damietta, Egypt,(2) assessing the antimicrobial resistance (AMR) patterns, including carbapenems and extended-spectrum β-lactamase genes; and (3) phylogenetically compare *Vibrio* isolates from shrimp and humans to evaluate zoonotic transmission risks.

## Materials and methods

### Sampling strategy

**Fig 1:**
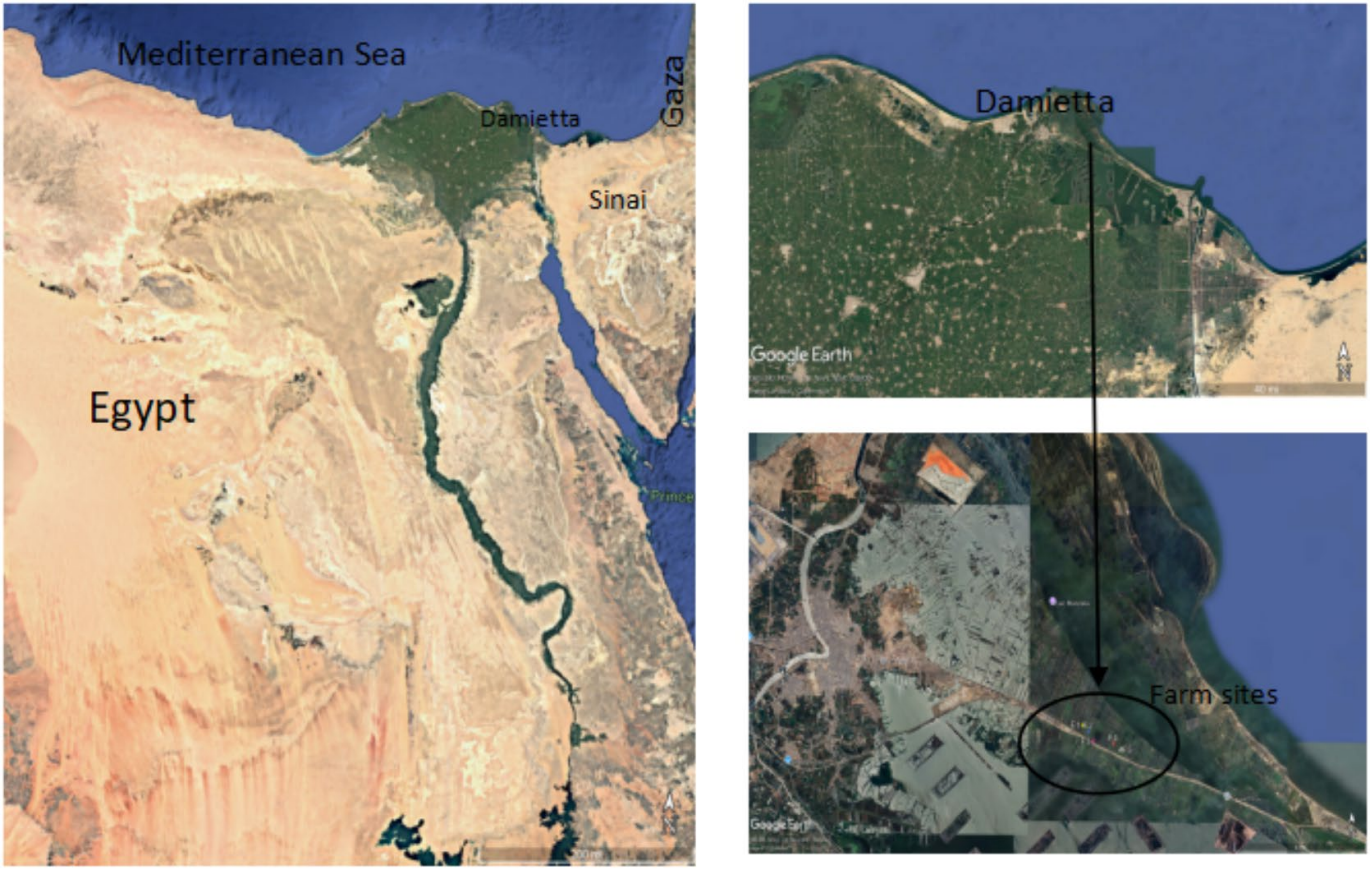
Map of sampling sites: the five shrimp farms in Damietta, Egypt. Satellite basemap imagery was obtained from Google Earth Pro (Version 7.3.6; Google LLC; https://www.google.com/earth). Labels were added using Microsoft Word (Version 2021; Microsoft Corporation). Imagery source: © 2026 Google, Maxar Technologies.

In the present study, fresh shrimp were caught after routine fishing. A cross-sectional study was conducted (December 2022 to February 2023) at five shrimp farms (Fig. 1), selected based on affected pond size, production system, and participation willingness. To recognize the key factors influencing productivity, sustainability, and potential interventions for improved aquaculture development, a structured survey willingly shared with farm owners was designed to gather data involving individual information and farm characteristics.

#### Shrimp samples

One hundred randomly collected *Penaeus indicus* (Indian Prawn) samples (20 specimens from each farm) were collected from the affected earthen ponds of the outbreaks occurred in December 2022–February 2023. Shrimp samples were transported alive in aerated plastic bags filled with pond water within 15 minutes to the Fish Diseases, Research, and Management Unit of the Damietta branch of Animal Health Research Institute, Agriculture Research Center (AHRI, ARC), Egypt, for further inspections. After arrival, samples were examined under aseptic conditions in a biological safety cabinet. Shrimp samples were stunned by ice, then surface sterilization was done with 70 % ethanol according to the International Commission on Microbiological Specifications for Food^30^. The length and weight of shrimp were measured. The average body weight and length of collected shrimp were 25-40 g and 13- 17 cm, respectively. Any visual signs were recorded, then the shrimp were dissected, and the hepatopancreas and muscle from each shrimp were aseptically removed, minced, and placed in alkaline peptone water (CM1028).

#### Water samples

Thirty water samples, 250 mL/each, were collected in a 500 mL sterile container from five different private shrimp farms (6 water samples/farm) according to the standard method described^31^. Water parameters pH and salinity were measured in situ using a pH meter (Lab Junction) and a refractometer (Brix, Diesella), respectively. The measured pH and salinity of the collected water samples during the study period ranged between 6.8–7.0 and 32–35 ppt, respectively. Collected water samples were labeled and transported in an icebox directly to AHRI, Damietta branch, for bacteriological examination.

#### Human samples collection

Thirty human stool samples were collected from fish farm workers who ate or handled shrimp from the selected farms and sent to the AHRI, Damietta branch laboratory. Informed verbal/written consent was obtained from the human participants. Demographic data of the sampled individuals were recorded, including health status, gender, and age group. Of the participants, 22 were diseased and 8 were apparently healthy. Human participants were classified as ‘diseased’ (n = 22) if they met the following criteria; ≥3 loose or watery stools per 24 hours; at least one of the following: abdominal cramps, nausea, vomiting, or documented fever ≥37.5°C; and no antibiotic use in the 14 days prior to sampling within the 7 days preceding stool sample collection, based on CDC^32^ case definitions for acute bacterial gastroenteritis. Apparently healthy’ participants (n = 8) reported no gastrointestinal symptoms in the 14 days prior to sampling and had no fever at the time of interview. No participant had a pre-existing laboratory-confirmed *Vibrio* diagnosis prior to enrollment,all *Vibrio* identifications were performed as part of this study. The case definition does not require microbiological confirmation a priori, as this study was designed to determine the etiological role of *Vibrio spp.* in symptomatic individuals^32^. The participants were comprised of 17 females and 13 males, with the following age distribution: 9 individuals were ≤10 years old, 8 individuals were 11-30 years old, and 13 individuals were >30 years old. All collected samples were kept refrigerated at 0 °C and were analyzed within 24 h. A summarized clinical symptom of the diseased participants was shown in Supplementary Table S1.

### Histopathological examination

Shrimp samples (hepatopancreas and muscle) were collected and examined for external gross signs of disease. For histopathological analysis, specimens from the most macroscopically affected shrimp samples were fixed in 10% (v/v) phosphate-buffered formalin (composed of 100 mL of 40% formalin, 900 mL distilled water, 4 g/L NaH₂PO₄, and 6.5 g/L Na₂HPO₄) for 24 hours. After fixation, the samples were preserved in 70% ethanol until further processing. The preserved specimens were then sent to the Veterinary Histopathology Lab (VHL), Faculty of Veterinary Medicine, Mansoura University (VMF-MU), Egypt, for tissue processing and embedding. Histopathological examination of shrimp was conducted blindly by a pathologist unaware of sample details, following Bancroft’s protocol for tissue processing and hematoxylin and eosin staining^33^. We used the G-grading system described by Lightner^34^ to quantify histological changes in shrimp infected with *Vibrio*. We assessed the severity of lesions (sloughing, necrosis, and inflammation) using a scale from 0 to 3, with 0 indicating normal tissue and 3 denoting significant damage.

### Phenotypic identification of *Vibrio* spp.

The isolation of *Vibrio* spp. was done in 2 steps; firstly, hepatopancreas and 10 g of shrimp flesh were homogenized separately, each in 90 mL of sterile alkaline peptone saline water (APSW, HiMedia, M618, India) and incubated at 35 ± 2 °C for 24-48 h^35^, whereas 50 mL of water samples were inoculated in 450 mL APSW. Human stool swabs were immersed in alkaline peptone water and incubated at 35 ± 2 °C for 24-48 h. A loopful of the enriched culture was streaked onto thiosulfate- citrate-bile salts-sucrose (TCBS) agar (CM0333, Oxoid), and the plates were incubated at 37 °C for 24 h^36^. Presumptive colonies (yellow and green) were then purified on TCBS and identified based on the morphological characteristics^37^ and biochemical examination^38^**.** Furthermore, the slide agglutination technique was applied for serotyping of *Vibrio* spp. using commercially available antisera kits (Denka Seiken Ltd., Tokyo, Japan) according to Wong et al.^39^, who recognized 13 O and 72 K types of commercial antisera. The phenotypic and biochemical characteristics of all identified species were confirmed according to Kaysner et al.^40^. Purified suspected *Vibrio* spp. colonies were stored in 70% glycerol at -20 ℃ until further molecular analysis.

### Antimicrobial susceptibility test

The antimicrobial susceptibility of the confirmed *Vibrio* phenotypes was carried out by Kirby-Bauer disc diffusion assay using commercial discs **(**Oxoid, Hampshire, UK) on Mueller-Hinton agar (HiMedia), and results were interpreted in accordance with the Clinical and Laboratory Standards Institute^41^^,^M100, 35^th^ edition) clinical breakpoints. Where available, epidemiological cut-off values (ECOFFs) for *Vibrio* spp. and relevant antimicrobial agents were also considered. In the absence of ECOFFs, CLSI (and/or EUCAST) clinical breakpoints were applied for interpretation. Fifteen antibiotic discs were selected based on their frequent usage in human and veterinary medicine^42,43^. These discs represent seven different antimicrobial classes: β-lactams (Penicillin: Ampicillin (AMP) 10μg,Cephalosporins: Cefotaxime (CTX) 30 μg, Ceftazidime (CAZ) 30 μg, Ceftriaxone (CRO) 30 μg, Cefoxitin (FOX) 30 μg; Carbapenems: Ertapenem (ETP) 10 μg, Meropenem (MRP) 10 μg); Aminoglycosides: Amikacin (AK) 30 μg; Fluorquinolones: Ciprofloxacin (CIP) 5 μg and Levofloxacin (LE) 5μg; Macrolides: Erythromycin (EO) 30μg and Azithromycin (AT) 15μg; Tetracycline: doxycycline (DO) 30μg; Sulfonamides: trimethoprim/sulfamethoxazole (COT) 1.25 μg/23.75 μg and Phenicols: Chloramphenicol (C) 30μg. *Escherichia coli* ATCC 25922 was used in accordance with CLSI recommendations, and that all susceptibility testing results for this strain fell within the acceptable quality control ranges. The multiple antibiotic resistance index (MAR) was measured as the ratio of the total number of antibiotics to which the *Vibrio* isolates displayed resistance to the number of tested antibiotics against *Vibrio* isolates^44^. If MAR is less than 0.2, this refers to low resistance (antibiotic use is likely controlled),meanwhile, if it is greater than 0.2, this means high resistance (indicates excessive antibiotic exposure).

To assess bacterial resistance trends, this study employs a standardized classification established by^45^. Multidrug-resistant (MDR): resistance strains to at least one agent from 3 or more classes,extensively drug-resistant (XDR): strains resistant to almost all antimicrobials; and pan-drug-resistant (PDR) strains: resistant strains to every tested antibiotic.

### Extraction of genomic DNA

Bacterial DNA was extracted from the phenotypically resistant, intermediate, and sensitive isolates using a conventional boiling method^46^. The NanoDrop Spectrophotometer was used to check the DNA concentration and purity of each sample. The extracted DNA was preserved at -20°C until further use in PCR.

Molecular detection of β-lactamase (ESBLs) and carbapenemase-encoding genes.

PCR for the detection of β-lactamase-encoding genes (ESBLs)

Multiplex polymerase chain reaction (PCR) was performed to detect the resistance determinant genes (TEM-type β-lactamase) *bla*_TEM_*,* (SHV-type β-lactamase) *bla*_SHV_*,* (Cefotaximase-Munich type) *bla*_CTX-M*,*_ and (Oxacillinase-1) *bla*_OXA-1_ using specific oligonucleotide primers set according to^47^ as listed in Table 1. PCR was performed following the method recorded by Mohammed et al.^48^.

**Table 1:** Primer sequences, target genes, amplicon sizes of ESBLs and Carbapenems’ genes.

**Genes**		**Primers sequences**	**Amp.** **Seg. (bp)**	**Ref.**
**β-lactamase-encoding genes (ESBLs)**	*bla*_TEM_	F: CGCCGCATACACTATTCTCAGAATGA R: ACGCTCACCGGCTCCAGATTTAT	445	^47^
	*bla*_SHV_	F: CTTTATCGGCCCTCACTCAA R: AGGTGCTCATCATGGGAAAG	237	
	*bla*_CTX-M_	F: ATGTGCAGYACCAGTAARGTKATGGC R: TGGGTRAARTARGTSACCAGAAYCAGCGG	593	
	*bla*_OXA-1_	F: ACACAATACATATCAACTTCGC R: AGTGTGTTTAGAATGGTGATC	813	
**carbapene-mase- encoding genes**	*bla*_KPC_	F: CATTCAAGGGCTTTCTTGCTGC R: ACGACGGCATAGTCATTTGC	538	^49^
	*bla*_VIM_	F: GATGGTGTTTGGTCGCATA R: CGAATGCGCAGCACCAG	390	
	*bla*_OXA-48_	F: GCTTGATCGCCCTCGATT R: GATTTGCTCCGTGGCCGAAA	281	
	*bla*_NDM_	F: GGTTTGGCGATCTGGTTTTC R: CGGAATGGCTCATCACGATC	621	^50^

Amp: amplified Seg: segment, Ref: reference

PCR for the detection of carbapenemase-encoding genes (bla_KPC_, bla_NDM,_ bla_VIM,_ and bla_OXA-48_)

Multiplex PCR to detect (*Klebsiella pneumoniae* carbapenemase) *bla*_KPC_ and (New Delhi metallo-β-lactamase) *bla*_NDM_ genes were performed using specific oligonucleotide primers set. An additional uniplex PCR test targeting the (Verona integron-encoded metallo-β-lactamase) *bla*_VIM_ and (Oxacillinase-48) *bla*_OXA-48_ genes were performed using specific oligonucleotide primers set^49,50^ as shown in Table 1. PCR was carried out according to the conditions and methodological details described by Mohammed et al.^48^.

All PCR products were separated on 1.5% agarose gel electrophoresis and visualized using a UV transilluminator. A 100 bp DNA ladder (100–1000 bp; Jena Bioscience GmbH, Germany) was used for size estimation of the amplicons. A no-template control (NTC), containing all PCR reagents except template DNA, was included in each run as a negative control. *Escherichia coli* ATCC 25922 and ATCC 43888 were used as positive quality control strains to verify the performance of the PCR assays for the detection of the target resistance genes.

Sequencing and phylogenetic analyses of the gyrB gene in vibrio fluvialis

Two biochemically confirmed *Vibrio fluvialis* isolates, one from a human and the other from shrimp hepatopancreas, were subjected to PCR amplification targeting the *gyrB* gene. The amplified fragments were purified using the QIAquick Gel Extraction Kit (QIAGEN, Germany) following the manufacturer’s protocol. Sequencing was performed with forward and reverse primers targeting the *gyrB* gene^51^. Sequence readings were analyzed using Sequencing Analysis Software and compared to the NCBI GenBank database using BLAST. A similarity of ≥ 99% was considered a successful identification. The resulting gene sequences were deposited in the National Center for Biotechnology Information (NCBI) GenBank database under accession numbers PV251882 and PV251883.

The sequenced *gyrB* genes were used for the genetic relationship assessment by comparing them with available *gyrB* sequences from clinical human isolates from the NCBI BLAST server, including critical care patients. The sequences of genes were retrieved from the NCBI GenBank and aligned with CLUSTALW implemented in the BioEdit version 7.0.1.4 and phylogenetic analysis was performed using MEGA version X. Robustness was given by a bootstrap consensus tree, built from 1000 replications.

### Statistical analysis

Shrimp sample size (n=100; 20 per farm) was determined based on expected prevalence of 70%^15^ to achieve a 95% confidence margin of error of ±9%^52^. Samples of water were taken as per standard aquaculture sampling guidelines by Austin et al.,^31^ (n=30,6 samples/farm). The fish farm employees who participated in the study formed a convenience sample (n=30) as all the consenting fish farm workers on the five farms were included (response rate 88.2%). In comparing diseased and healthy participants, a post hoc power analysis (G*Power 3.1,^53^ showed 80% power when using a large effect size (w=0.50). Both R (Version 4.4.3) and PASW Statistics (Version 18.0,SPSS Inc., Chicago, IL, USA) were used for statistical analysis. A nested structure was observed in the data and samples were potentially clustered amongst different farms, so a Generalized Linear Mixed Model (GLMM) was used^54^. In this model the individual sample was used as the unit of analysis. The treatment of Sample Source (Shrimp Hepatopancrease, Shrimp Muscle, Water, and Human Stool) was treated as a fixed effect and the treatment of ‘Farm ID’ was considered as random effect to account for intra-farm correlation and environmental variance. Overall, the association of the sources with the prevalence of *Vibrio* was assessed by the Type II Wald chi-square test using the car package^55^. Odds Ratios (OR) were computed using the fixed effects of the model to show the probability of recovery of *Vibrio* spp. compared to the baseline level in humans. Fisher’s Exact Test was used for species with low frequency (N<5 in a cell).

The association between demographic data of humans and the prevalence of *Vibrio* spp. was tested by the Chi-square test and the Cramer’s *V* test, where ≤ 0.2 indicates a weak association, > 0.2 to ≤ 0.6 indicates a moderate association, and > 0.6 indicates a strong association. The significance level (*p*-value) was set at the 0.05 level. Heatmaps were generated by the ‘*heatmap’* package^56^.

### Biosecurity measures

Biosecurity measure was taken according to the recommendations of the Public Health Agency of Canada^57^. All isolation activities were conducted with personal protective equipment (PPE) (face mask, gloves, lab coat) and strict hand hygiene measures were taken with alcohol-based sanitizer and strong antiseptics. After each use equipment was cleaned and all samples disposed of hygienically (incinerated).

## Results

### Characteristics of the examined shrimp farms

The relationship between farm characteristics and the prevalence rate of *Vibrio* spp. assessed via a structured questionnaire in Table 2. The surveyed shrimp farms were operated by male owners aged 51-58 years, all holding a diploma-level education. Farm owners had excellent to good experience levels in shrimp farming. Farms varied in size (2-5 feddans) and production type (semi-intensive to intensive), all utilizing pond systems. Higher stocking densities (Farm 3: 30,000/F) correlated with elevated mortality (35%) in the affected ponds, while lower densities of the affected ponds in shrimp farms (Farm 4: 10,000/F) showed better survival (10-20%) in the examined affected pond. Veterinary supervision (Farms 3 & 4) appeared to reduce mortality compared to unsupervised farms. Total production rates fluctuated between 0.5 and 1 ton per feddan in all farm ponds, with market weights averaging 25-40 g. Antibiotics and growth promoters were rarely used, but probiotics were applied in two farms (Farms 1 & 2). Water exchange frequency and probiotic use may influence shrimp health, warranting further investigation.

**Table 2:** The key characteristics of the examined shrimp farms.

	**Key factors**	**Farm 1**	**Farm 2**	**Farm 3**	**Farm 4**	**Farm 5**
**Farm owners’ features**	**Age**	58	55	57	51	52
	**Gender**	Male	Male	Male	Male	Male
	**Edu. Level**	Diploma	Diploma	Diploma	Diploma	Diploma
	**Experience** **level**	Excellent	excellent	Very good	Very good	good
**Farm features**	**Production type**	Semi intensive	Intensive	intensive	intensive	intensive
	**Farm size (Fd)**	2 Fd	5 Fd	5 Fd	2 Fd	3 Fd
	**Prod. methods**	Pond sys.	Pond sys.	Pond sys.	Pond sys.	Pond sys.
	**Stock den. / F**	50000/F	20000/F	30000/F	10000/F	20000/F
	**Water source**	ML, MD	ML, MD	ML, MD	ML, MD	ML, MD
	**Veterinary supervision**	No	No	Yes	Yes	No
	**Use poultry manure**	No	No	No	No	No
	**Own equipment**	Yes	Yes	Yes	Yes	Yes
	**Disinfect. Equip.**	PS	PS	No	No	No
	**Feed store**	Yes	Yes	Yes	Yes	Yes
	**Water exchange rate**	10-20%, p/d	10-20%, p/d	Daily (ND)	Daily (ND)	sometimes
	**Pond water quality**	Turbid	turbid	Turbid	turbid	turbid
	**Test water quality**	Once weekly	Once weekly	Daily	Daily	Weekly
	**Feeding management**	Apd	Apd	Apd	Apd	Apd
	**Disinfection**	BS	BS	BS	BS	NA
**selected pond** **features**	**Pond size**	1200 m^2^	1100 m^2^	1000 m^2^	1200 m^2^	1100 m^2^
	**stock den. /Pond**	15000	20000	25000	18000	22000
	**Disposal of dead shrimp**	In	In	In	In	In
**Shrimp**	**Mor. R.**	50%	60%	35%	10-20%	40%
	**Mor. RL2y**	40-50%	40%	30%	20%	40%
	**Prod. R.**	1 ton/ Fd	1 ton/Fd	0.5 ton/Fd	0.75/Fd	0.55/Fd
	**Market wt.**	25-30 gm	40 gm	40 gm	30 gm	40 gm
	**Antibiotics**	No	No	YES	No	No
	**Growth promotors**	No	No	NO	No	No
	**Probiotics**	Yes	Yes	NO	No	No
	**Clin. S./R.**	Fragile shell/occasionally	Red shell / occasionally	White and red spots / occasionally	White and red spots/ occasionally	Decreased growth / occasionally

F: farm, Fd: feddan (Area unit used in Egypt equals 4200 m^2^), ML: El-manzala Lake, MD: Mediterranean Sea, PS: Physical disinfection (sunrays), p/d: of pond daily, Apd: artificial pelleted diet, BS: before stocking, NA: not applied, In: incineration, Mor. R.: mortality rate in the examined affected pond, Mor. RL2y: mortality rate last 2 years, Prod. R: production rate, wt.: weight, Clin. S./ R: clinical signs/ rate, sys: system, Shrimp samples (N = 100; 20 per farm); Water samples (N = 30; 6 per farm).

### Examination of shrimps

**Fig. 2:**
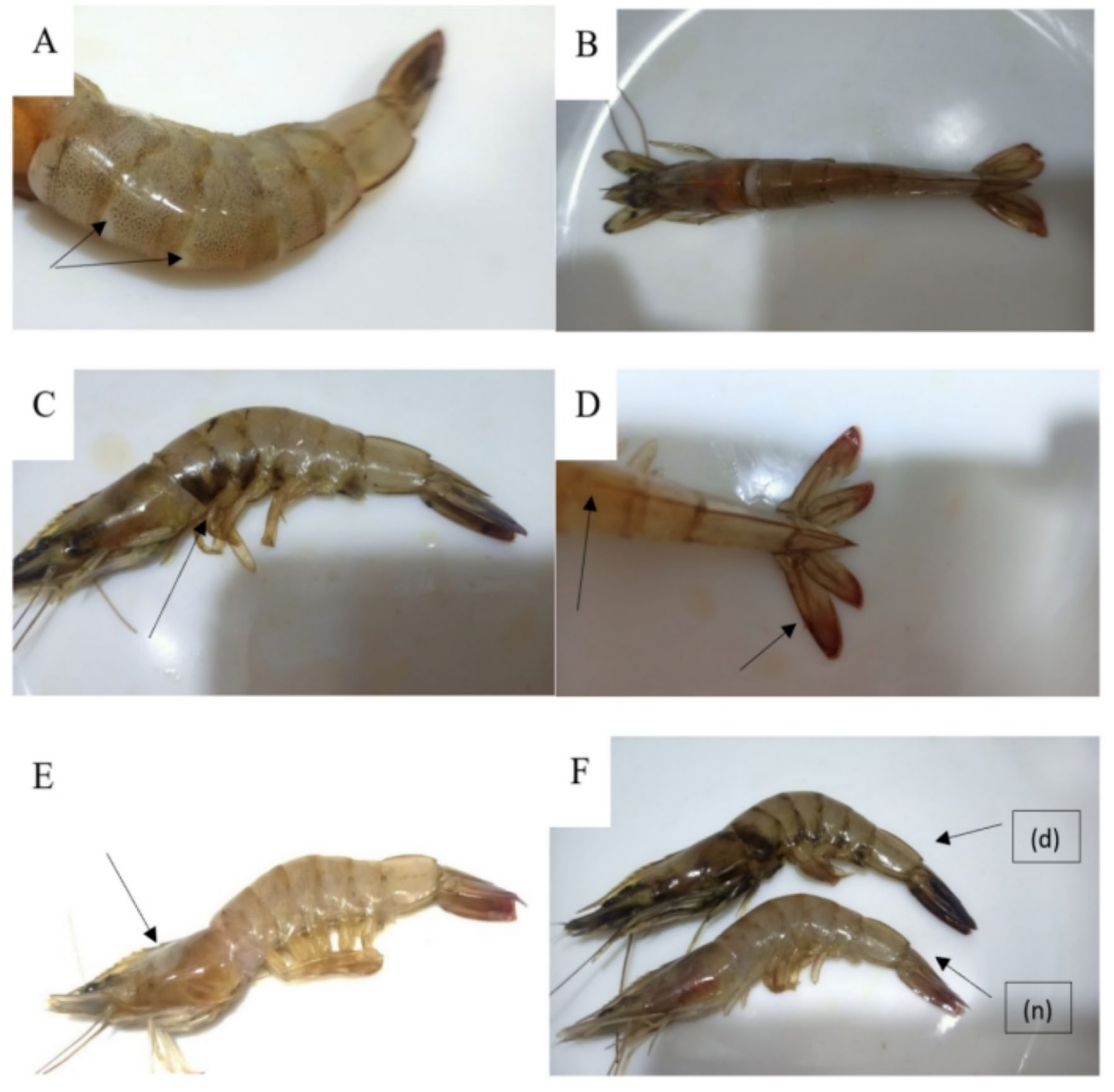
Clinical signs of *Vibrio* spp. infection in shrimp; a: white focal coloration in muscles, b: opacity, red discoloration in the body, c: melanosis on the cuticle, d: empty intestine, redness and black coloration in tail, e: paleness in hepatopancreas and f: hyper melanosis of diseased shrimp (d) versus normal shrimp (n).

**Fig. 3:**
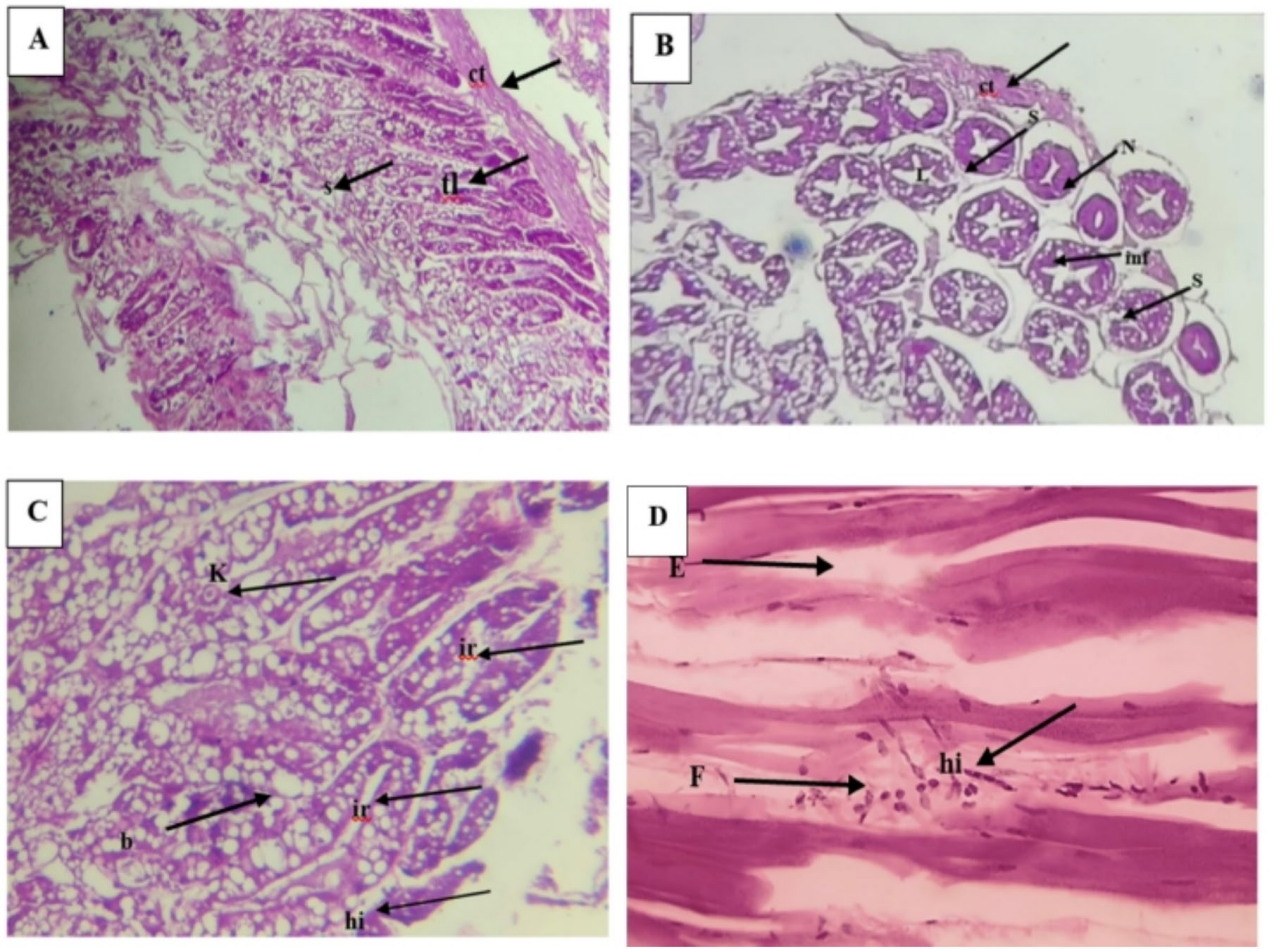
Microscopic pictures of diseased shrimp using semi-quantitative scoring; hepatopancreas (**A**, **B** and **C**) and muscle (**D**) showing sloughing of epithelial cells of hepatopancreas (S) (scored 1), ir: irregular hepatopancreatic lumen, K: karyomegaly, hi: hemocytic infiltration (scored 1-2), b: transverse section of B cell with subapical vacuoles dispersed in most of the cytoplasm and basal nucleus (scored 1-3 ), N: necrosis, inf: infiltration, L: lumen, ct: connective tissue, tl: tubular lumen, D: longitudinally striated skeletal muscle showing E: edema and F: fibrosis with hi: hemocytic infiltration.

The examination of the collected shrimp revealed that shrimp that were naturally infected displayed outward symptoms and postmortem lesions that were indicative of bacterial infection (Fig. 2). Lethargy, an empty stomach and midgut, and a pale to white atrophied hepatopancreas were noticed with hemorrhagic spots. Clinical septicemia was evident in the shrimps. The majority of shrimp had reddening, blackening around the tail, legs, and shell. Others showed signs of body color fading, fragile shells, shell redness, white/red spots, melanization and muscle white spots. Fig. 3 shows diseased shrimp hepatopancreas with sloughed epithelial cells, irregular hepatopancreatic lumen, karyomegaly, hemocytic infiltration and dispersed vacuoles in most of the cytoplasm and basal nucleus. Longitudinal sections of striated skeletal muscle of diseased shrimp exhibited oedema, fibrosis, and hemocytic infiltration within the muscle tissue.

### The prevalence of *Vibrio* spp. among examined farms

From December 2022 to February 2023, a qualitative analysis was conducted to detect *Vibrio* species in 100 shrimp and 30 water samples collected from five shrimp farms as listed in Table 3. The overall prevalence of *Vibrio* in shrimp and water samples was 90% (90/100) and 56.7% (17/30), respectively in the affected ponds of each farm. All farm affected ponds exhibited high *Vibrio* prevalence rates, ranging from 75% to 100%. Farm 1 had the lowest recorded rate (75%), whereas Farm 5 had the highest record (100%). Similarly, water samples from Farm 5 had the highest *Vibrio* prevalence rate (83%), while the other farms showed lower frequencies: 66.7% (Farm 4), 50% (Farms 2 and 3), and 33.3% (Farm 1).

**Table 3:** Proportion of *Vibrio* species recorded from examined farms.

**Sample type**	**Farms**					**Total**
	**1**	**2**	**3**	**4**	**5**	
**Shrimp (n= 100)**	15 (75%) *	17 (85%)	19 (95%)	19 (95%)	20 (100%)	90/100 (90%)
**Water (n= 30)**	2 (33.3%) **	3 (50%)	3 (50%)	4 (66.7%)	5 (83%)	17/30 (56.7%)
**Total**	65.4%***	76.9%	84.6%	88.5%	96.2%	82.3%

Shrimp samples (20/ farm), Water samples (6/farm). *Percentage was calculated according to no. of infected shrimp to total no. of shrimp in the examined farm pond. ** percentage was calculated according to no. of positive water samples to total no. of water samples in the examined farm pond *** percentage was calculated according to no. of positive samples (shrimp and water) to total no. of (shrimp and water) samples in the examined farm pond.

**Fig. 4:**
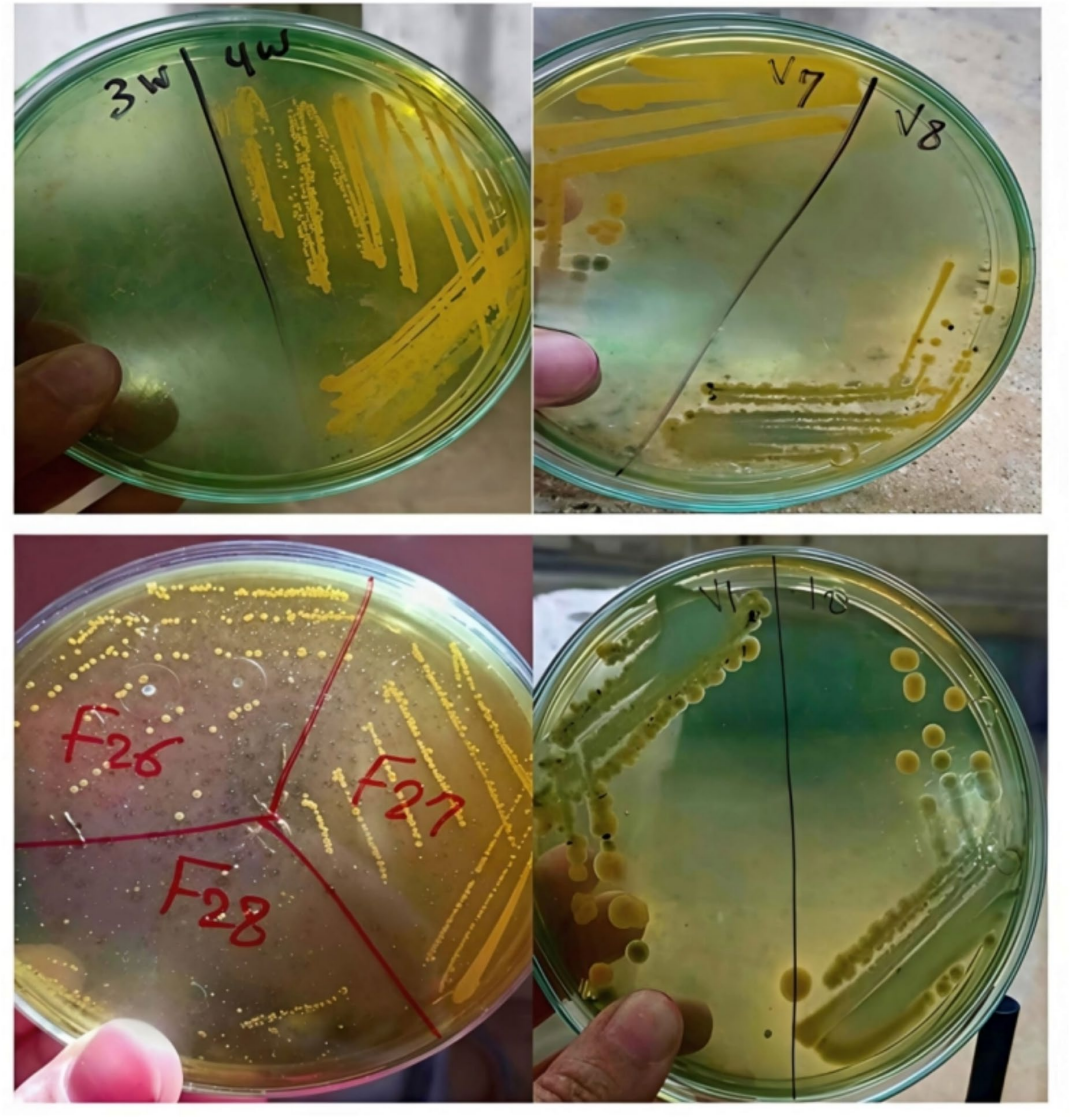
Different *Vibrio* spp. colonies on TCBS media (yellow & green colonies).

*Vibrios* spp. were isolated from TCBS agar plates. In contrast to *V. cholera*, *V. alginolyticus*, and *V. fluvialis*, which were smooth and yellow (sucrose +ve), typical colonies of *V. parahaemolyticus*, *V. vulnificus*, and *V. mimicus* showed smooth and green (sucrose -ve) (Fig. 4). Gram negative-stained films revealed that *Vibrio* species were comma-shaped, short, stiff, Gram-negative, and with a single flagellum.

#### Prevalence and distribution of *Vibrio* spp. across shrimp, water, and human stool samples

In Table 4, the analysis revealed some interesting differences in how often *Vibrio spp*. showed up in various sample types. Shrimp hepatopancreas had the highest contamination rate at 90.0%, followed by water at 56.7%, shrimp muscle at 48.0%, and human stool at 33.3%. There was a strong link between the type of sample and the overall *Vibrio* infection. The association between sample source and *Vibrio* prevalence was analyzed using a Generalized Linear Mixed Model (GLMM) to account for the clustering of samples within farms (random effect). The analysis revealed a highly significant effect of sample source on *Vibrio* detection (*χ*^2^= 38.78, *df* = 3, adjusted *p* < 0.0001). Post-hoc analysis using the reference group (human stool) demonstrated that shrimp hepatopancreas had the strongest association with *Vibrio* presence, yielding an odds ratio (OR) of 23.50. Conversely, water samples and shrimp muscle displayed ORs of 2.81 and 1.88, respectively.

**Table 4:** Prevalence of *Vibrio* spp. in naturally infected shrimp, water, and human samples.

***Vibrio* spp*.***	**Total Positive (%)**				
	**Shrimp hepatopancreas** **(n=100)**	**Shrimp Muscle** **(n=100)**	**Water** **(n=30)**	**Human Stool** **(n=30)**	**Adjusted** ***p*-value**
**Total infected**	90 (90.0)	48 (48.0)	17 (56.7)	10 (33.3)	<0.0001*
***V. mimicus***	3 (3.0)	7 (7.0)	1 (3.3)	0	0.602 (*FET*)
***V. fluvialis***	39 (39.0)	17 (17.0)	6 (20.0)	6 (20.0)	0.004*
***V. parahaemolyticus***	10 (10.0)	2 (2.0)	1 (3.3)	0	0.147
***V. damsela***	0	1 (1.0)	0	1 (3.3)	0.864 (*FET*)
***V. vulnificus***	3 (3.0)	2 (2.0)	1 (3.3)	0	0.969 (*FET*)
***V. furnissi***	3 (3.0)	2 (2.0)	0	0	0.977 (*FET*)
***V. alginolyticus***	28 (28.0)	16 (16.0)	5 (16.7)	3 (10.0)	0.087
***V. cholera***	4 (4.0)	1 (1.0)	2 (6.7)	0	0.474 (*FET*)
***V. holliseae***	0	0	1 (3.3)	0	1.000 (*FET*)

Statistical significance was evaluated using Generalized Linear Mixed Models (GLMM), with ‘Farm ID’ included as a random effect to account for the hierarchical clustering of samples within individual farms.

For species with low frequency (<5 positive cases per source), Fisher’s Exact Test (*FET*) was employed.

Adjusted *p*-values represent the source-effect significance after controlling for intra-farm environmental variance.

Statistically significant results (*p* < 0.05) are marked with an asterisk (*) and **bolded**.

Regarding species-specific patterns, *V. fluvialis* was the most frequently common isolate, that appeared in shrimp hepatopancreas (39.0%), shrimp muscle (17.0%), and water and human stool samples (20.0%) (adjusted *p* = 0.004). Furthermore, *V. parahaemolyticus* was found in shrimp hepatopancreas at 10.0%, but sporadic in other samples, showing up at 3.3% or less (adjusted *p* = 0.147). *V. alginolyticus* was spread in shrimp, with 28.0% in hepatopancreas and 16.0 % in muscle, although it didn’t show significant differences among sample types (adjusted *p* = 0.087). The less common species such as *V. mimicus, V. damsela, V. vulnificus, V. furnissi, V. cholera,* and *V. holliseae* were found to be quite rare, with prevalences less than 10% and with no significant associations (adjusted *p* > 0.05), except for *V. mimicus* in shrimp muscle, which was found at 7.0%.

### Prevalence and risk factors of *Vibrio* infections across demographic and clinical groups of humans

Table 5 depicted that *Vibrio* spp. infection occurred in 33.3% of the examined population. Infection status was clearly linked with other factors such as health status, gender, and even age, although the patterns varied by species. It was obvious that the prevalence of infection in those with health issues had a 45.5%, while people who appeared healthy showed no infections at all (Cramer’s *V* = 0.43, *p* = 0.020). All *Vibrio* isolates were recovered from diseased humans presenting with gastrointestinal complaints and diarrhea, and each patient was infected with a single *Vibrio* species. When looking at gender differences, females had a higher infection rate of 47.1% compared to males at 15.4%, although this difference was only marginally significant (Cramer’s *V* = 0.33, *p* = 0.068). In terms of age, the highest infection rate was found in individuals over 30 years old at 53.8%, followed by those aged 10 years or younger at 22.2%, and those between 11 and 30 years at 12.5%, but no clear differences were found (Cramer’s *V* = 0.39, *p* = 0.104). Looking at species-specific prevalence, *V. fluvialis* was the most found species at 20.0%, followed by *V. alginolyticus* at 10.0% and *V. damsela* at 3.3%. A moderate, non-significant association was found for individual species (Cramer’s *V* = 0.22, *p* = 0.118).

**Table 5:** Prevalence and risk factors of *Vibrio* infections across demographic and clinical groups of human samples.

**Variables**	**Categories**	**Total**	**Positive**	**%**	**Cramer’s *V***	***p*-value**
***Total Infection***		30	10	33.3		
***Health status***	**Apparently healthy**	8	0	0	**0.43**	0.020*
	**Diseased**	22	10	45.5		
***Gender***	**Female**	17	8	47.1	0.33	0.068
	**Male**	13	2	15.4		
***Age group***	**≤10 years**	9	2	22.2	0.39	0.104
	**11 – 30 years**	8	1	12.5		
	**> 30 years**	13	7	53.8		
***Vibrio* spp.**	***V. fluvialis***	30	6	20.0	0.22	0.118
	***V. damsel***		1	3.3		
	***V. alginolyticus***		3	10.0		

Cramer’s *V* values were used to assess the strength of association between *Vibrio* species prevalence and sample types. A Cramer’s *V* ≤ 0.2 indicates a weak association, > 0.2 to ≤ 0.6 a moderate association, and > 0.6 a strong association. Significance was set at *p* < 0.05. Statistically significant results are marked with (*) **bolded**.

### Serological identification of *Vibrio parahaemolyticus*

Serological examination of *V. parahaemolyticus* isolates (supplementary data) revealed the abundance of O5: k17 serotype (30.8%), followed by O11: K50 (23.1%) and O8: K39 (15.4%). Serotypes O1: K38, O4: K13 and O10: K52 accounted for 7.7% and one untypeable species.

### The antibiotic susceptibility profile of *Vibrio* isolates

The investigation of 40 *Vibrio* isolates demonstrated a significant incidence of antimicrobial resistance as shown in Table 6. Most of the isolates (31/40, equating to 77.5%) manifested resistance to three or more classes of antimicrobials, thereby categorized as multi-drug resistant (MDR) or extensively drug-resistant (XDR). A minimal level of drug resistance, pronounced by resistance to one or two drugs (MAR index: 0.13), was detected in merely 6 isolates (15%), while (MAR index: 0.20), was detected in 3 isolates (7.5%). These resistance patterns predominantly involved Erythromycin (EO) in conjunction with various other antimicrobial agents.

**Table 6:** *Vibrio* serovar classification based on antimicrobial resistance phenotype and MAR index (n=40).

**Resistance pattern**^**1**^	**Antimicrobial Resistance phenotype**	**No. and % of *Vibrio* spp.**	**Phenotypes**^**2**^	**MAR index (a/b)**^**3**^	**Classification of strains**	
					**Type of resistance**	No. & (%)* of strains
**I**	EO- COT	1 (2.5%)	*v. fluvialis* (1)	0.13	Low drug resistant	9 (22.5%)
	AK- EO	2 (5%)	*v. alginolyticus* (1) *v.fluvialis* (1)	0.13		
	CTX- EO	2 (5%)	*v. fluvialis* (2)	0.13		
	AMP- EO	1 (2.5%)	*v. alginolyticus* (1)	0.13		
**II**	MRP- ETP- EO	3 (7.5%)	*v. fluvialis* (3)	0.20		
**III**	CRO- MRP- ETP- EO	2 (5%)	*v. parahaemolyticus* (1) *v. mimicus* (1)	0.27	Multi-drug resistant (MDR)	23 (57.5%)
	CAZ- MRP- AT- C	2 (5%)	*v. cholera* (1) *v. alginolyticus* (1)	0.27		
	AMP- CTX- CAZ- EO	1 (2.5%)	*v. alginolyticus* (1)	0.27		
**IV**	AMP- CTX- CAZ- EO- COT	3 (7.5%)	*v. fluvialis* (2) *v. parahaemolyticus* (1)	0.33		
	AMP- CAZ- AT- EO- COT	1 (2.5%)	*v. alginolyticus* (1)	0.33		
	AMP- CTX- CAZ- EO- COT	1 (2.5%)	*v. alginolyticus* (1)	0.33		
	CTX- MRP- ETP- AT- EO	1 (2.5%)	*v. fluvialis* (1)	0.33		
	FOX- CAZ- MRP- ETP- EO	6 (15%)	*v. fluvialis* (5) *v. alginolyticus* (1)	0.33		
**V**	AMP- CTX- CAZ- MRP- ETP- EO	2 (5%)	*v. alginolyticus* (1) *v. damsela* (1)	0.40		
	AMP- FOX- CTX- CRO- CAZ- EO	1 (2.5%)	*v. fluvialis* (1)	0.40		
**VI**	AMP- CTX- MRP- ETP- AT- EO- COT	2 (5%)	*v. mimicus* (1) *v. vulnificus* (1)	0.46		
	AMP- CTX- CRO- CAZ- EO- DO- CIP	1 (2.5%)	*v. vulnificus* (1)	0.46		
**VII**	AMP- CTX- CAZ- MRP- ETP- AK- EO- C	4 (10%)	*v. fluvialis* (1) *v. furnissi* (2) *v. parahaemolyticus* (1)	0.53	Extensively-drug resistant (XDR)	8 (20%)
**VIII**	AMP- CTX- CAZ- MRP- ETP- EO- DO- CIP- COT	1 (2.5%)	*v. mimicus* (1)	0.60		
	CTX- CRO- CAZ- MRP- ETP- AK- EO- COT- C	1 (2.5%)	*v. vulnificus* (1)	0.60		
	AMP- CTX- CAZ- AK- EO- DO- CIP- COT- C	1 (2.5%)	*v. mimicus* (1)	0.60		
**IX**	AMP- CTX- CAZ- MRP- ETP- EO- DO- CIP- COT- C	1 (2.5%)	*v. parahaemolyticus* (1)	0.66		

^1^EO= Erythromycin COT= trimethoprim/sulfamethoxazole, AK= Amikacin, CTX= Cefotaxime, AMP= Ampicillin, ETP= Ertapenem, CRO= Ceftriaxone, MRP= Meropenem, CAZ= Ceftazidime, AT= Azithromycin, C= Chloramphenicol, FOX= Cefoxitin, DO= doxycycline, CIP= Ciprofloxacin and LE= Levofloxacin. ^2^*Vibrio* isolates are classified related to their antimicrobial resistance phenotypes. ^3^MAR (Multiple Antibiotic Resistance) index was calculated for each *Vibrio* strain using formula A/B (where A is the number of antimicrobials to which an isolate was resistant, and B is the total number of antimicrobial agents examined according to^58^. *The percentage of the resistance strains was calculated as the number of resistance strains to total number of examined isolates (n=40).

Twenty-three *vibrio* isolates (57.5%) as the most prevalent category, classified as multi-drug resistance (MDR) strains showing 12 unique resistance patterns, with MAR indices ranging from 0.27 to 0.46. The most frequently identified MDR pattern was FOX-CAZ-MRP-ETP-EO, which was detected in 6 isolates (15.0%), *V. fluvialis* and *V. alginolyticus* were the predominant serotypes within the MDR cohort.

Markedly, 8 isolates (20.0%) were categorized as extensively drug-resistant (XDR), presenting MAR indices between 0.53 and 0.66 and evocating resistance to nearly all tested antimicrobial agents. Different resistance patterns appeared were distributed among several serotypes, including *V. furnissii*, *V. parahaemolyticus*, and *V. mimicus*. The most pronounced resistance pattern (MAR index: 0.66), which showed resistance to ten distinct antimicrobials, was identified in a singular *V. parahaemolyticus* isolate.

**Fig. 5:**
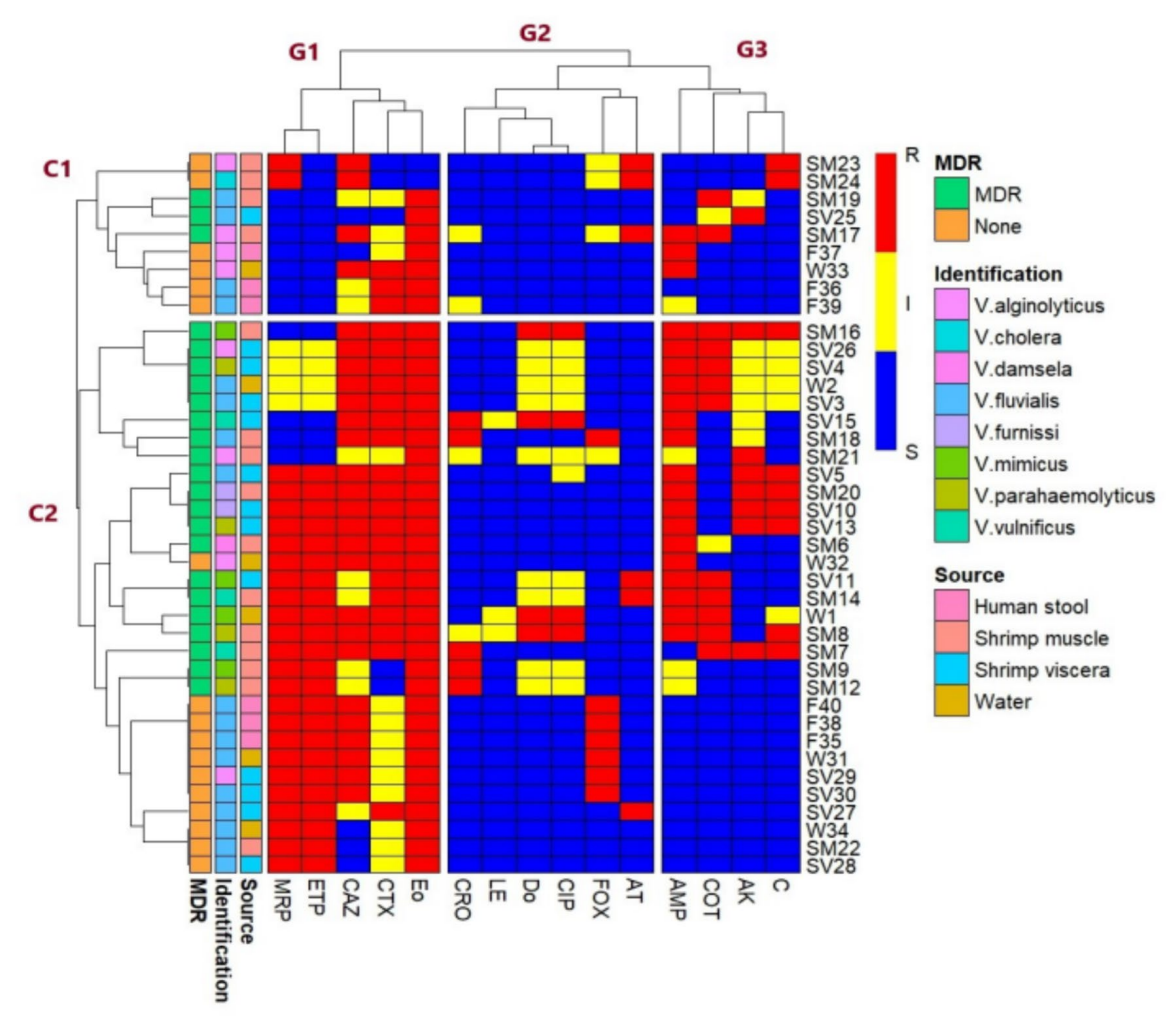
Clustered heatmap showing *Vibrio* isolates’ phenotypic profile. The phenotypic pattern was displayed as follows: Resistant (R, red), Intermediate (I, yellow), and Sensitive (S, blue). The source of samples was coded as: F (Human stool), SM (Shrimp muscle), SV (Shrimp hepatopancreas), and W (Water). Antibiotic abbreviations: AMP: Ampicillin; FOX: Cefoxitin; CTX: Cefotaxime; CRO: Ceftriaxone; CAZ: Ceftazidime; MRP: Meropenem; ETP: Ertapenem; AK: Amikacin; AT: Azithromycin; EO: Erythromycin; DO: Doxycycline; CIP: Ciprofloxacin; LE: Levofloxacin; COT: Trimethoprim-sulfamethoxazole; C: Chloramphenicol.

In Fig. 5, the clustered heatmap of *Vibrio* isolates’ phenotypic profile was shown. It displays a heatmap showing the phenotypic antibiotic susceptibility profiles of *Vibrio* isolates, with hierarchical clustering based on resistance pattern similarities. Two major clusters of isolates (C1 and C2) were identified. Cluster C1 comprised a smaller subset of isolates, predominantly from shrimp muscle samples, which exhibited susceptibility to most antibiotics except erythromycin (EO) and ceftazidime (CAZ). Cluster C2 involved the majority of isolates, from various sources, including human stool (F), shrimp hepatopancreas (SV), shrimp muscle (SM), and water (W). These isolates showed higher resistance, especially to EO, cefotaxime (CTX), ceftazidime (CAZ), ertapenem (ETP), and meropenem (MRP). Antibiotics were also grouped based on their overall effectiveness; Group G2 included the most effective antibiotics; azithromycin (AT), cefoxitin (FOX), ciprofloxacin (CIP), doxycycline (DO), levofloxacin (LE), ceftriaxone (CRO); Group G3 consisted of antibiotics with intermediate resistance; chloramphenicol (C), amikacin (AK), trimethoprim-sulfamethoxazole (COT), ampicillin (AMP); and Group G1 comprised antibiotics to which the isolates showed the highest resistance; erythromycin (EO), cefotaxime (CTX), Ceftazidime (CAZ), Ertapenem (ETP) and Meropenem (MRP).

### Molecular identification of *Vibrio* spp.

**Fig. 6:**
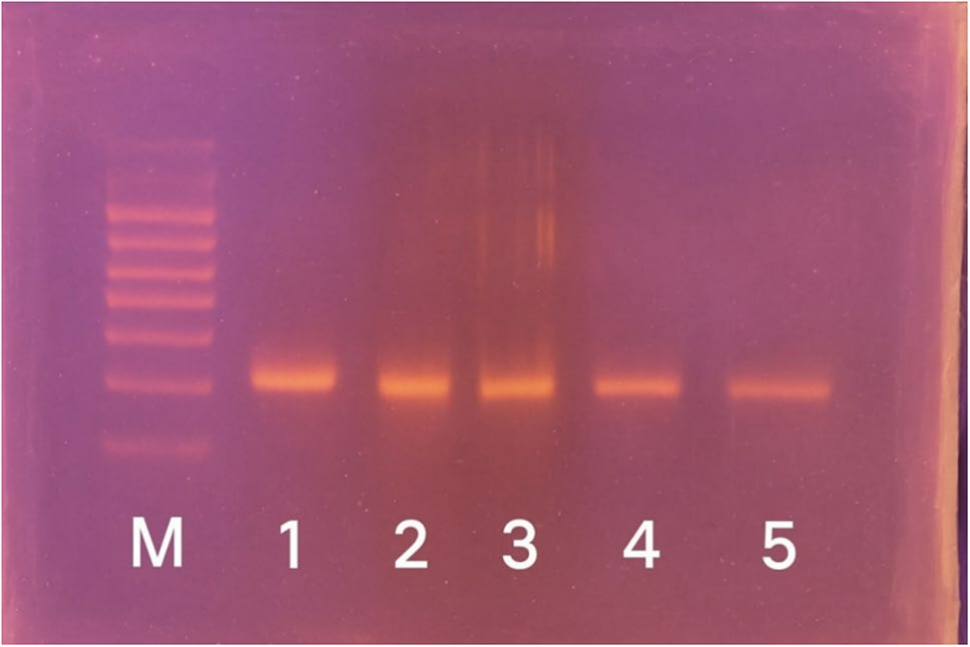
The amplified bands of resistance genes, A; La.ne M: Marker, Lane 1: positive control, lanes 2-5 *bla*_*SHV*_ positive isolates.

**Fig. 7:**
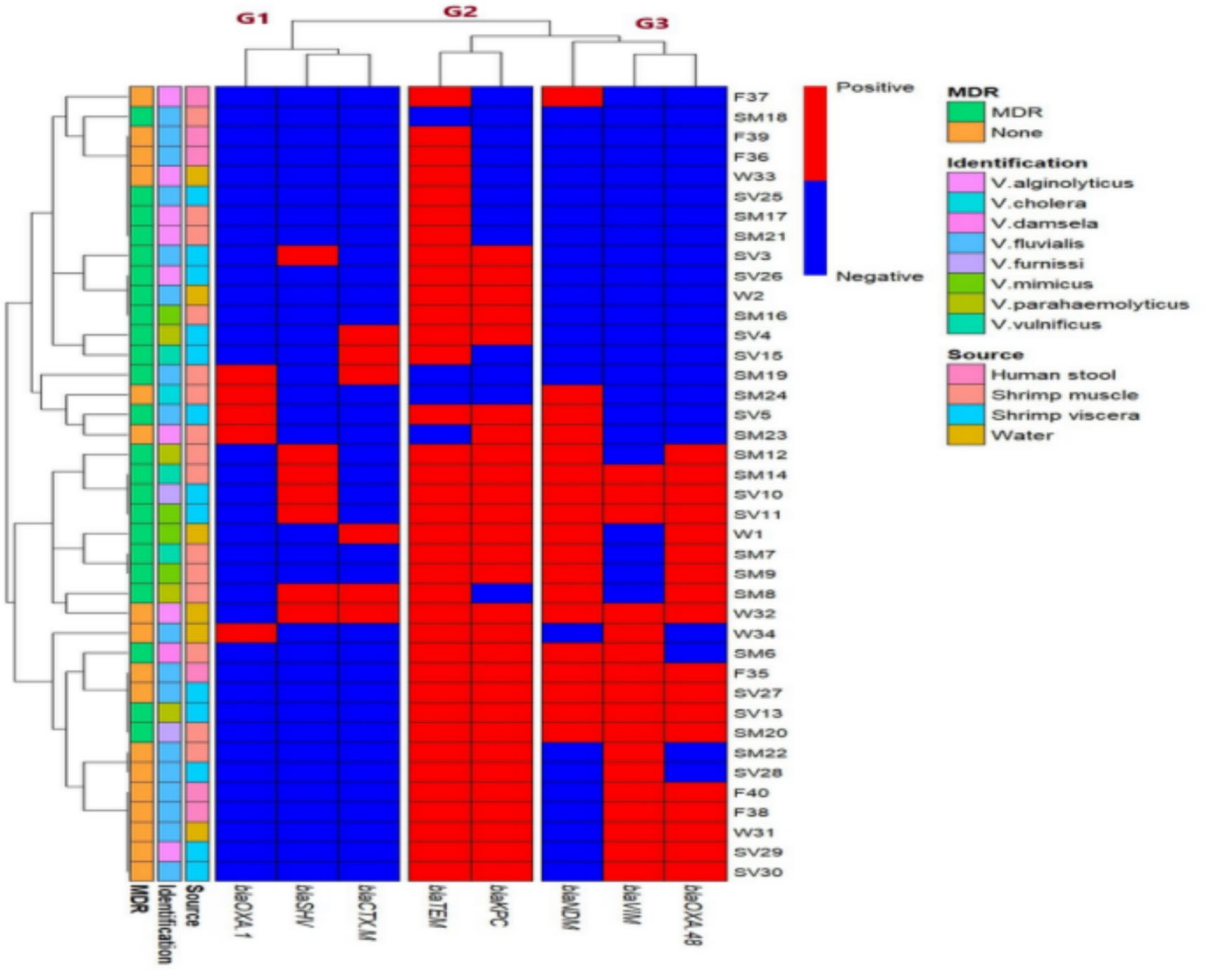
Clustered heatmap showing *vibrio* isolates’ genotypic resistance profile. The presence of β-lactamase-encoding genes was screened, including *bla*_*TEM*_ (TEM-type β-lactamase), *bla*_*SHV*_ (SHV-type β-lactamase), *bla*_*CTX-M*_ (Cefotaximase-Munich type), *bla*_*OXA-1*_ (Oxacillinase-1), and carbapenemase-encoding genes including *bla*_*VIM*_ (Verona integron-encoded metallo-β-lactamase), *bla*_*OXA-48*_ (Oxacillinase-48), *bla*_*NDM*_ (New Delhi metallo-β-lactamase), and *bla*_*KPC*_ (Klebsiella pneumoniae carbapenemase) was screened. Isolates exhibiting resistance to three or more antimicrobial classes were classified as MDR (multidrug-resistant). The genotypic resistance was displayed as: Positive (Red) and Negative (Blue). The source of samples was coded as: F (Human stool), SM (Shrimp muscle), SV (Shrimp hepatopancrease), and W (Water).

The identified *Vibrio spp*. isolates (n=40) were subjected to PCR for the detection of antibiotic resistance genes (Fig. 6). The results showed that 90%, 17.55%, 15%, and 12.5% of the isolates under investigation had beta-lactamase genes, the *bla* (*TEM*, *SHV*, *CTX-M*, and *OXA-1*) genes, respectively. Additionally, in this investigation, carbapenem genes represented by *bla* (*VIM*, *OXA-48*, *NDM*, and *KPC*) were found in 42.5%, 45%, 45%, and 70% of the samples that were analyzed, respectively (Fig. 7). The clustered heatmap showed the genotypic resistance profile, illustrating the distribution of β-lactamase-encoding genes among the isolated *Vibrio* isolates.

Resistance genes were divided into three groups according to their overall prevalence, with Group G2 being the most prevalent genes (*bla*_*TEM*_ and *bla*_*KPC*_), Group G3 the intermediate prevalence genes (*bla*_*OXA-48*_, *bla*_*VIM*_, and *bla*_*NDM*_), and Group G1 the less prevalent genes (*bla*_*CTX-M*_, *bla*_*SHV*_, and *bla*_*OXA-1*_). Almost half of the *Vibrio* isolates contained 3–4 resistance genes. Multidrug-resistant (MDR) isolates were mainly found in Groups G2 and G3, and were frequently co-produced with other resistance determinants, including *bla*_*TEM*_, which is also likely to be involved in MDR. Remarkably, there was no restriction of the distribution of resistance determinants to a single sample source, as MDR isolates were found in the majority of samples collected.

**Fig. 8:**
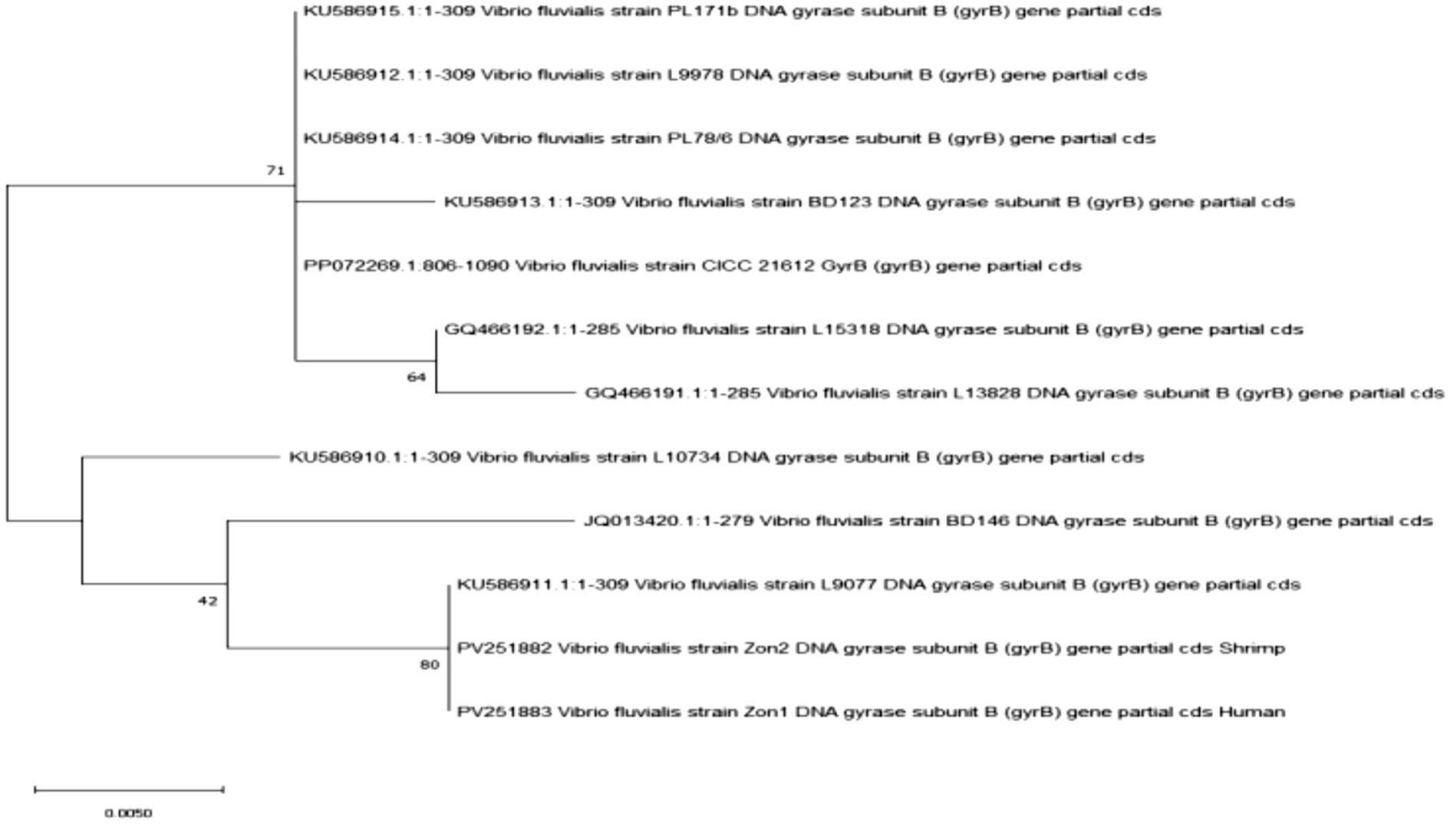
Phylogenetic relationships based on *gyrB* gene sequences of selected isolates. The trees were constructed and analyzed by the Maximum Likelihood with 1000 bootstrap.

Phenotypic and genotypic resistance profile of *Vibrio* isolates (n=40) was added in supplementary data Table 2. In addition, the isolate-level concordance was added in supplementary data Table S3. Comparing the *gyrB* gene sequences revealed 100% nucleotide identity between the selected isolate from shrimp hepatopancreas (accession PV251882) and the human isolate (accession PV251883), as shown in Fig. 8. The *gyrB* gene is a reliable marker for species-level identification in *Vibrio*, but it lacks the discriminatory power for strain-level transmission inference.

## Discussion

One of the major serious complex threats that cause mass mortality in marine aquaculture is *vibrio*. The wide phylogenetic diversity and opportunistic traits of *Vibrio* genus confirmed that vibriosis is a multifaceted disease rather than just a simple infection^59^. For years, the accurate scale of this hidden diversity has been obscured by conventional Standard diagnostics. Though, advanced genomic technologies are now uncovering a plethora of novel species that were previously misidentified or utterly unknown^60^.

The intensification of shrimp production heightens stress on aquatic organisms, leading to the rise of diseases that severely affect business^61^. The pathogens affecting the shrimp are viral, bacterial, fungal and parasitic. Notably, white spot syndrome virus (WSSV) stands first in causing serious economic loss followed by bacterial diseases which are estimated to cause a loss of over USD 6 billion to the shrimp farming industry^62^. Among the bacterial pathogens affecting the shrimp, *Vibrio* remains the single most important pathogen causing huge economic losses to the aquaculture industry^63^. Detailed information may be sought on Vibriosis associated with fish and marine fish^64^,*Vibrios* in seafood^65^,AMR in *Vibrio parahaemolyticus* in seafood^66^ and aquaculture^67^.

Markedly, this current study sheds light on the key operational factors that influence *Vibrio*-related mortality in shrimp farms of the studied area. The correlation of higher stock density with higher mortality rates indicates that elevated density might result in greater stress in birds and raise the risk of pathogen transmission. Signs observed in the shrimp samples which include shell discoloration, fragility atrophied hepatopancreas and lesions are similar to those observed during vibriosis, confirming its importance in shrimp production.

One of main findings that we have come across during our study is the positive correlation of the number of farms and the prevalence of *Vibrio*. This can be attributed to the shared source of water in the farms. The overall prevalence in sample farms (82.3%) was seen and there was a difference between types of samples. The shrimp samples had a higher detection rate (90%) than water samples (56.7%): showing that the bacteria are actively hosted in the shrimp samples. A clear trend where the prevalence increased from affected ponds of Farm 1 to Farm 5 in both types of samples was noticed. This pattern implies that there are additional underlying causes such as farming practices or environmental conditions, which could be causing the risk of transmission and the low water parameters, this is in accordance with^68^.

Our findings reveal a clear hierarchy of risk, shrimp hepatopancreas samples were the primary reservoir for *Vibrio* spp., with a surprising prevalence (90%), signifying a food safety issue. Although the muscle tissue that people consume was less contaminated, almost half of it still contained. The high statistical correlation (p<0.0001) between the sample type and the infection confirms that the source is important. The detection of *V. fluvialis* and *V. parahaemolyticus* pathogens known to cause gastroenteritis in both the shrimp and the human stool samples, suggesting that there is a probable path of transmission in between the shrimp and human in contact. These findings collectively point to shrimp and aquaculture water as the key points in the pathogenic *Vibrio* species transmission cycle.

The reason behind the high infection rates in shrimp farms is that shrimp can often be exposed to contaminated sewage filled with bacteria because the shrimp lives in muddy habitats^69^. This environment creates an ideal condition in which harmful pathogens such as *V. parahaemolyticus*, *V. fluvialis*, and *V. vulnificus* could increase causing significant losses in aquaculture over the years^70,71,72^. Additionally, *Vibrio alginolyticus*, *Vibrio vulnificus*, and *Vibrio mimicus* were detected in the dying shrimp samples, with the respective percentages 25.58, 22.09, and 17.44%. This suggests that they might have a role in the observed shrimp death^9^. Even more prevalence rates were found in our research as compared to some regional research, for instance, Azwai et al.^73^, which reported a rate of 52.3%. This highlights the importance of local biogeochemical factors such as soil, water quality, and biosafety measures, which affect the type of *Vibrio* populations present in each farm, and hence affect the number of infections and mortality events.

The *vibrio* infections may be accelerated by close contact between aquacultures. This may be because of the significant amount of untreated domestic, commercial and agricultural drainage water that enters El-manzala Lake every year. Elgendy et al.^9^ tentatively suggested that the *Vibrio* infection was due to transport of the sediment in the water column by the sea waves and the significant winds in the Mediterranean region during the study period.

In this study, Farm 5 showed the highest viable *Vibrio* count in its water (83%), this directly relates to its significantly higher shrimp infection rate. This assumes that water quality is an important infection determinant. *Vibrio* distribution varied from place to place with the distribution of species reported by Heenatigala and Fernando^74^ showing differences in the distribution of specific *Vibrio* species. Farming management has played a major role in this variability, and often the poor quality of the water, typically caused by poorly planned canal systems, allows the spread of pathogens as farm water flows from one to another. Furthermore, the difference in fish and water infection rate can be explained by the origin of fish fry, and the source of the strain^75^.

Given the omnipresence of this environment, infection in humans is an important public health issue. This study assumes the spillover issue by finding *Vibrio* spp. in 33.3 % of the diarrhoeic human stool samples, indicating the possible role of *Vibrio* spp. as enteric pathogen in the area. Interestingly, *V. fluvialis* (20%) was the prevalent common species, which is in line with its newly recognized worldwide ubiquity.

This has implications on human health that are not-trivial, particularly for opportunistic pathogens like *V. vulnificus* that can be acquired from contaminated seafood or water and lead to necrotizing fasciitis. The distribution of species in our study (*V. fluvialis*, *V. alginolyticus, V. damsela*) is varied from the percentages of other parts of the world^76^. This heterogeneity is in fact acknowledged and has been blamed on geographical, climatic and methodological reasons^75^. These infections are then enabled by the same environmental and infrastructural problems mentioned earlier,poor sanitation, warm water and poor handling practices that start a transmission chain from farm to consumer^77^, which is an urgent need for integrated monitoring from the aquaculture site to the consumer.

One of the limitations of this study is that the sample period was limited to the winter season in Egypt (December 2022 – February 2023). There are seasonal patterns in the abundance of *Vibrios*, with higher concentrations usually observed in water temperatures >20°C (May – October) as reported by Baker-Austin et al.,^8^. Our prevalence estimates (90% in shrimp, 33% in humans) therefore do not necessarily represent the annual average and are likely to underestimate the risk for transmission during the warm season. The occurrence of carbapenemase genes and MDR phenotypes, though, was a clinically important discovery as it happened during the winter, a time when *Vibrio* prevalence is expected to be at its lowest, suggesting year-round carriage. Multiple seasonal samplings should be included in future research to describe annual *Vibrio* cycles. This study was a study of an outbreak during a period of mortality that was observed (December – February) and its results are therefore applicable to the period of the epidemic^78^.

All 13 *V. parahaemolyticus* isolates’ serotypes were examined, and the results showed that 12 *V. parahaemolyticus* isolates had different O and K antigens. The results revealed that all isolates taken from the same farm showed different serotypes. The isolates are thought to have descended from different *V. parahaemolyticus* clones, which disagree with Kongrueng et al.^79^ who reported identical *Vibrio* species serotypes.

Antibiotic resistance is one of the biggest public health security concerns confronting humanity in the twenty-first century, according to the World Health Organization^80^. Antibiotic resistance (ABR) is a global threat with significant economic and public health consequences, with 1.27 million deaths attributed to ABR globally^81^. In vivo studies utilizing animal models to assess antibiotic sensitivity to *Vibrio* infections may not be universally applicable to people due to variations in pharmacokinetic characters^82^. According to reports, aquatic creatures only consume 20–30% of antibiotics, with the remaining 70–80% ending up in the environment, supposed a relationship between environmental pollution in aquaculture and antibiotic resistance, which will increase during pollution and could transmit to the surroundings^83^.

Common antibiotics used in aquaculture, including ampicillin, gentamycin, oxytetracycline, chloramphenicol, trimethoprim, and kanamycin, were shown to be ineffective against all the *Vibrio* isolates^74^.

Antimicrobial resistance (AMR) profiling reveals a high resistance prevalence among the isolated *Vibrio* spp., that considers serious interest in public health and aquaculture management. A significant proportion of the strains (20%) were classified as extensive drug resistance (XDR) and a large percentage (57.5%) were multi-drug resistant (MDR), with extensive resistance phenotypes being observed in different species such as *V. fluvialis*, *V. alginolyticus* and *V. parahaemolyticus*. The high MAR levels, many >0.2, confirm exposure to high pollution environments where antibiotics use is probably high and indicate a very serious one health problem: emergence of resistance in aquaculture practice may compromise the effectiveness of therapeutic interventions in human medicine. In this study, the patterns of antibiotic resistance of *Vibrio* spp. isolates were determined and an alarming extent of multi-drug resistance (MDR) was discovered. Thirty-one subjects (77.5%) were resistant to 3 or more classes. The increase in resistance is probably associated with poor use of antibiotics in aquaculture.

In terms of effectiveness, levofloxacin was found to be the most effective antimicrobial agent, showing 92.5% susceptibility. This confirms its recommendation to be the recommended treatment in the field, which is in line with the results of^84^. Fluoroquinolone class, on the other hand, has a complicated finding: it has a high efficacy in this context, but it is also associated with a decreased mortality rate in vibriosis^76^. However, it is controversial because of its known health risks^85^, and because it is not approved for aquaculture in the U.S.^86^. Low resistance rates for the two fluoroquinolones, ciprofloxacin (10%) and levofloxacin (0%), might be due to this regulatory restriction.

Furthermore, the results of the research showed considerable resistance to antibiotics that are commonly used. An example is the high level of resistance to erythromycin (95%), and some cephalosporins like ceftazidime (65%). Moreover, beta-lactams resistance was also notable, with half of the isolates resistant to ampicillin, this aligns with the increased presence of beta-lactam resistance genes in aquaculture environments^87^. *Enterobacteriaceae* were once more commonly resistant to carbapenems, but this is now a concern in *Vibrio* spp.^26^. These genes are probably acquired via mobile genetic elements^88^

The spread of antibiotic resistance genes (ARGs) is an overwhelming one health challenge as the genetic elements are no longer contained in clinical practices. ARGs have been detected in wetlands, and aquaculture systems^89^. The gut microbiome of the animals in aquaculture is thought to be the main repository of plasmid-encoded multidrug-resistant ARGs, which can readily be passed between the bacteria inhabiting the gut^90^.

Some of the most problematic ARGs that are encoding resistance to a crucial class of antibiotics are beta-lactamases and enzymes. The use of antibiotics in human medicine and agriculture is a strong driver of the spread of such genes^91^. Our results correspond with this worldwide threat, with the presence of beta-lactamase genes in high prevalence of the *Vibrio* spp. isolates. Most importantly, 90% of isolates carried the *bla*_*TEM*_ gene and other genes including *bla*_*SHV*_ (17.5%), *bla*_*CTX-M*_ (15%) and *bla*_*OXA-1*_ (12.5%) were also found. On the contrary, Rahman et al.^92^ identified *bla*_CARB_ as a key gene in *Vibrio*, suggesting possible geographic or host-specific alterations in the carriage of resistance gene.

A particularly alarming finding is the high detection rate of carbapenems’ genes, that confer resistance to last-resort antibiotics. The present study found that *bla*_KPC_, *bla*_NDM_, *bla*_OXA-48_, and *bla*_VIM_ were present in 70%, 45%, 45%, and 42.5% of isolates, respectively. This explains the findings by Walsh et al.^93^ who reported that carbapenem resistance, once primarily associated with *Enterobacteriaceae*, is increasingly emerging in non-fermenters like *Vibrio* spp. Furthermore, reports for *bla*_NDM_ (45%) coincide with the trend identified by Igere et al.^94^^,^ who found the NDM-1 gene in 32.85% of aquatic *V. cholerae*.

The subpopulation of isolates in this study was phenotypically non-carbapenem resistant, but harbored carbapenemase genes. The difference in genotypic and phenotypic resistance has been well documented in environmental *Vibrio* spp. and is explained by various mechanisms. First, gene presence does not mean functional expression; carbapenemase genes might be silent because of promoter mutations or insertion sequence disruptions^95^. Second, heteroresistance, the presence of resistant subpopulations below the threshold of detection by disk diffusion, has been reported in gram-negative bacteria^96^. This phenomenon is particurarly relevant, as the presence of *bla*_*NDM*_ and *bla*_*KPC*_ has been previously described in aquatic *Vibrio* isolates^26,49^. Third, the expression of carbapenemases can be modulated by the environment, as there is a difference between the natural habitat of the environment and standard lab conditions^11,27^. Furthermore, Hamza et al.^97^ and Igere et al.^94^ have observed similar discordances in *V. fluvialis* from Nile Tilapia and *V. cholera* from aquatic resources, respectively. Carbapenems are thus the best practice marker for resistance potential, but phenotypic confirmation is recommended for clinical risk assessment, by means of MIC testing.

The difference between genetic susceptibility and physical resistance is well documented in environmental *Vibrio* spp. and can be attributed to several factors. First, a gene alone does not necessarily determine its function; these genes can be “silent”, because of mutations in the promoter or disruption of its structure^95^. Second, aquatic *Vibrio* that carry *bla*_*NDM*_ and *bla*_*KPC*_ may have been found in tiny resistant subpopulations which are not detected by standard tests (known as heteroresistance), as mentioned by El-Halfawy and Valvano^96^ and Goh et al.,^26^. Also, factors in the environment, such as salinity and temperature, can affect gene expression differently than it does in a controlled laboratory environment^11, 27^. These mismatches are also observed by Hamza et al.^97^ and Morris et al.^98^. One interesting incident was the finding of a complete *bla*_*NDM-1*_ gene in imported prawns from which *V. alginolyticus* was isolated, that was still considered sensitive to meropenem. This resulted in them thinking that *Vibrio* could be “hidden reservoirs” that only express resistance genes under specific conditions^98^. These marine microorganisms can transfer resistance to other bacteria^99^, which represents a threat to human health. Therefore, identification of these genes is an important early warning, but we recommend the use of MIC testing for more precise clinical risk assessment. Even though our isolates are expressing *bla*_*NDM*_ at low levels, it is easy to imagine the gene being transferred to more clinically relevant hosts, giving a high likelihood of resistance emerging in the future.

Eventually, the difference in the pattern of resistance development across the present study and others, like the variance in particular beta-lactamase genes, underscores the importance of the environmental pressures and host factors in the selection of resistance, a concept supported by Mohamed et al.^100^. The ubiquitous nature of ARGs in the environment demonstrates an urgent need for integrated surveillance and antimicrobial stewardship measures that encompass both human health and environmental management.

The observed 100% sequence homology in the *gyrB* gene between selected shrimp hepatopancreas isolates and human isolates may suggest a possible link between strains from different hosts, as reported in the literature. The *gyrB* gene has been well documented as a species-level molecular marker for *Vibrio* and is especially useful when phenotypic techniques are constrained by the variability of the organisms^51^. But even if *gyrB* is a housekeeping gene that is conserved among species, this alone is not enough to prove cross-species transmission. Thus, the results are to be used with some caution. More discriminatory methods like multilocus sequence typing (MLST) or whole genome sequencing (WGS) are needed to give more robust support to the transmission dynamics. Additionally, the MAR index threshold of 0.2 was originally validated for fecal *E. coli*^44^ and lacks species-specific validation for aquatic *Vibrio*. However, this index remains a useful comparative tool for identifying high-risk resistance patterns across studies.

## Conclusion

The present investigation highlights a clear relationship between the poor farm hygiene and pandemic vibriosis in *Penaeus indicus*, which was further compounded by the high prevalence (more than 75%) of MDR *Vibrio* isolates with cephalosporinase and carbapenemase genes. The results of our study, especially the genetic similarity of shrimp and human isolates, indicate a big One Health threat. They offer a much-needed evidence base that is critical for the urgent paradigm shift needed in the Egyptian shrimp aquaculture industry, from reacting to antibiotic use to implementing proactive, ecological management. We suggest immediate implementation of improved biosecurity measures, farmer education and the use of sustainable alternatives, such as probiotics, in order to ensure industry sustainability and public health. Ecological therapeutics need to be developed in the future and the relationship between *Vibrio* virulence, antimicrobial resistance and climate change needs to be explained.

## Supplementary Information

Supplementary Information.

## Data Availability

All data supporting the findings of this study are available on paper.
