# Supplementary material for: Multidrug-Resistant Vibrio spp. carrying carbapenemase genes in aquacultured shrimp and their zoonotic importance
Source: Sci Rep. 2026 Jul 22;16:22955. doi: 10.1038/s41598-026-62098-x (PMC13392438; doi:10.1038/s41598-026-62098-x)
Supplement: Supplementary file 1 — Supplementary Information. [file 41598_2026_62098_MOESM1_ESM.docx]

**Table S1. Clinical symptoms reported by diseased human participants (n = 22) before stool sample collection**

| Symptom | Number of Participants (n = 22) | Percentage (%) |
| --- | --- | --- |
| Watery diarrhea (≥3 episodes/24 hours) | 22 | 100% |
| Abdominal cramps | 19 | 86.4% |
| Fever (documented ≥37.5°C) | 15 | 68.2% |
| Nausea | 12 | 54.5% |
| Vomiting | 9 | 40.9% |

**Notes:** All 22 participants met the clinical case definition for acute bacterial gastroenteritis (WHO, 2017; CDC COVIS surveillance case definition). Participants could report multiple symptoms. No participant reported bloody diarrhea. Data were derived from structured interviews and clinical records at the time of stool collection (December 2022 – February 2023).

**Table S2. Phenotypic and genotypic resistance profile of *Vibrio* isolates (n=40)**

| **Source** | **Biochemical identification** | **Phenotypic pattern** | | | | | | | | | | | | | | | ***Genotypic pattern*** | | | | | | | | **MDR** |
| --- | --- | --- | --- | --- | --- | --- | --- | --- | --- | --- | --- | --- | --- | --- | --- | --- | --- | --- | --- | --- | --- | --- | --- | --- | --- |
|  |  | **penicillin** | **cephalosporin** | | | | **carbapenema** | | **amin** | **macrolids** | | **tetra** | **fluro** | | **trime** | **pheni** | ***b lactamase*** | | | | ***carbapenemas*** | | | |  |
|  |  | AMP | FOX | CTX | CRO | CAZ | MRP | ETR | AK | AT | EO | DO | CIP | LE | COT | C | ***bla_TEM_*** | ***bla_SHV_*** | ***bla_CTX-M_*** | ***bla_OXA.1_*** | ***bla_VIM_*** | ***bla_OXA.48_*** | ***bla_NDM_*** | ***bla_KPC_*** |  |
| W | *Vv. mimicus* | R | S | R | S | R | R | R | S | S | R | R | R | I | R | I | + | - | + | - | - | + | + | + | √ |
| W | *V. fluvialis* | R | S | R | S | R | I | I | I | S | R | I | I | S | R | I | + | - | - | - | - | - | - | + | √ |
| V | *V. fluvialis* | R | S | R | S | R | I | I | I | S | R | I | I | S | R | I | + | + | - | - | - | - | - | + | √ |
| V | *V. parahaemolyticus* | R | S | R | S | R | I | I | I | S | R | I | I | S | R | I | + | - | + | - | - | - | - | + | √ |
| V | *V. fluvialis* | R | S | R | S | R | R | R | R | S | R | S | I | S | S | R | + | - | - | + | - | - | + | + | √ |
| M | *V. damsela* | R | S | R | S | R | R | R | S | S | R | S | S | S | I | S | + | - | - | - | + | - | + | + | √ |
| M | *V. vulnificus* | S | S | R | R | R | R | R | R | S | R | S | S | S | R | R | + | - | - | - | - | + | + | + | √ |
| M | *V. parahaemolyticus* | R | S | R | I | R | R | R | S | S | R | R | R | I | R | R | + | + | + | - | - | + | + | - | √ |
| M | *V. mimicus* | I | S | S | R | I | R | R | S | S | R | I | I | S | S | S | + | - | - | - | - | + | + | + | √ |
| V | *V. furnissi* | R | S | R | S | R | R | R | R | S | R | S | S | S | S | R | + | + | - | - | + | + | + | + | √ |
| V | *V. mimicus* | R | S | R | S | I | R | R | S | R | R | I | I | S | R | S | + | + | - | - | + | + | + | + | √ |
| M | *V. parahaemolyticus* | I | S | S | R | I | R | R | S | S | R | I | I | S | S | S | + | + | - | - | - | + | + | + | √ |
| V | *V. parahaemolyticus* | R | S | R | S | R | R | R | R | S | R | S | S | S | S | R | + | - | - | - | + | + | + | + | √ |
| M | *V. vulnificus* | R | S | R | S | I | R | R | S | R | R | I | I | S | R | S | + | + | - | - | + | + | + | + | √ |
| V | *V. vulnificus* | R | S | R | R | R | S | S | I | S | R | R | R | I | S | S | + | - | + | - | - | - | - | - | √ |
| M | *V. mimicus* | R | S | R | S | R | S | S | R | S | R | R | R | S | R | R | + | - | - | - | - | - | - | + | √ |
| M | *V. alginolyticus* | R | I | I | I | R | S | S | S | R | R | S | S | S | R | S | + | - | - | - | - | - | - | - | √ |
| M | *V. fluvialis* | R | R | R | R | R | S | S | I | S | R | S | S | S | S | S | - | - | - | - | - | - | - | - | √ |
| M | *V. fluvialis* | S | S | I | S | I | S | S | I | S | R | S | S | S | R | S | - | - | + | + | - | - | - | - | √ |
| M | *V. furnissii* | R | S | R | S | R | R | R | R | S | R | S | S | S | S | R | + | - | - | - | + | + | + | + | √ |
| M | *V. alginolyticus* | I | I | I | I | I | S | S | R | S | R | I | I | S | S | S | + | - | - | - | - | - | - | - | √ |
| M | *V. fluvialis* | S | S | I | S | S | R | R | S | S | R | S | S | S | S | S | + | - | - | - | + | - | - | + | - |
| M | *V. alginolyticus* | S | I | S | S | R | R | S | S | R | S | S | S | S | S | R | - | - | - | + | - | - | + | + | √ |
| M | *V. cholera* | S | I | S | S | R | R | S | S | R | S | S | S | S | S | R | - | - | - | + | - | - | + | _ | √ |
| V | *V. fluvialis* | S | S | S | S | S | S | S | R | S | R | S | S | S | I | S | + | - | - | - | - | - | - | - | √ |
| V | *V. alginolyticus* | R | S | R | S | R | I | I | I | S | R | I | I | S | R | I | + | - | - | - | - | - | - | + | √ |
| V | *V. fluvialis* | S | S | R | S | I | R | R | S | R | R | S | S | S | S | S | + | - | - | - | + | + | + | + | √ |
| V | *V. fluvialis* | S | S | I | S | S | R | R | S | S | R | S | S | S | S | S | + | - | - | - | + | - | - | + | - |
| V | *V. alginolyticus* | S | R | I | S | R | R | R | S | S | R | S | S | S | S | S | + | - | - | - | + | + | - | + | √ |
| V | *V. fluvialis* | S | R | I | S | R | R | R | S | S | R | S | S | S | S | S | + | - | - | - | + | + | - | + | √ |
| W | *V. fluvialis* | S | R | I | S | R | R | R | S | S | R | S | S | S | S | S | + | - | - | - | + | + | - | + | √ |
| W | *V. alginolyticus* | R | S | R | S | R | R | R | S | S | R | S | S | S | S | S | + | + | + | - | + | + | + | + | √ |
| W | *V. alginolyticus* | R | S | R | S | R | S | S | S | S | R | S | S | S | S | S | + | - | - | - | - | - | - | - | √ |
| W | *V. fluvialis* | S | S | I | S | S | R | R | S | S | R | S | S | S | S | S | + | - | - | + | + | - | - | + | - |
| F | *V. fluvialis* | S | R | I | S | R | R | R | S | S | R | S | S | S | S | S | + | - | - | - | + | + | + | + | √ |
| F | *V. fluvialis* | S | S | R | S | I | S | S | S | S | R | S | S | S | S | S | + | - | - | - | - | - | - | - | - |
| F | *V. alginolyticus* | R | S | I | S | S | S | S | S | S | R | S | S | S | S | S | + | - | - | - | - | - | + | - | - |
| F | *V. fluvialis* | S | R | I | S | R | R | R | S | S | R | S | S | S | S | S | + | - | - | - | + | + | - | + | √ |
| F | *V. fluvialis* | I | S | R | I | I | S | S | S | S | R | S | S | S | S | S | + | - | - | - | - | - | - | - | - |
| F | *V. fluvialis* | S | R | I | S | R | R | R | S | S | R | S | S | S | S | S | + | - | - | - | + | + | - | + | √ |

^1^EO= Erythromycin COT= trimethoprim/sulfamethoxazole, AK= Amikacin, CTX= Cefotaxime, AMP= Ampicillin, ETP= Ertapenem, CRO= Ceftriaxone, MRP= Meropenem, CAZ= Ceftazidime, AT= Azithromycin, C= Chloramphenicol, FOX= Cefoxitin, DO= doxycycline, CIP= Ciprofloxacin and LE= Levofloxacin. F: human stool, W: water, V: shrimp hepatopancrease, M: shrimp muscle

**Table S3. Isolate-level concordance between carbapenemase gene carriage and phenotypic carbapenem resistance by disk diffusion**

| Isolate ID | Source | Species | *bla*_KPC | *bla*_NDM | *bla*_OXA-48 | *bla*_VIM | MRP Phenotype | ETP Phenotype | Concordance |
| --- | --- | --- | --- | --- | --- | --- | --- | --- | --- |
| VF-SV-01 | Shrimp hepatopancreas | *V. fluvialis* | + | - | - | - | R | R | Yes |
| VF-SV-02 | Shrimp hepatopancreas | *V. fluvialis* | + | - | - | - | R | I | Partial |
| VF-SV-03 | Shrimp hepatopancreas | *V. fluvialis* | + | - | - | - | I | S | No |
| VF-SV-04 | Shrimp hepatopancreas | *V. fluvialis* | + | - | - | - | R | R | Yes |
| VF-SV-05 | Shrimp hepatopancreas | *V. fluvialis* | + | - | - | - | I | I | Partial |
| VF-SV-06 | Shrimp hepatopancreas | *V. fluvialis* | - | + | - | - | S | I | No |
| VF-SV-07 | Shrimp hepatopancreas | *V. fluvialis* | + | - | - | - | R | R | Yes |
| VF-SV-08 | Shrimp hepatopancreas | *V. fluvialis* | - | + | + | - | I | S | No |
| VF-SV-09 | Shrimp hepatopancreas | *V. fluvialis* | + | - | - | - | S | I | No |
| VF-SV-10 | Shrimp hepatopancreas | *V. fluvialis* | - | - | - | + | S | S | No |
| VA-SV-11 | Shrimp hepatopancreas | *V. alginolyticus* | + | - | - | - | R | R | Yes |
| VA-SV-12 | Shrimp hepatopancreas | *V. alginolyticus* | - | + | - | - | I | I | Partial |
| VA-SV-13 | Shrimp hepatopancreas | *V. alginolyticus* | + | - | - | - | R | I | Partial |
| VA-SV-14 | Shrimp hepatopancreas | *V. alginolyticus* | - | - | + | - | S | S | No |
| VP-SV-15 | Shrimp hepatopancreas | *V. parahaemolyticus* | + | + | - | - | S | S | No |
| VP-SV-16 | Shrimp hepatopancreas | *V. parahaemolyticus* | + | - | - | - | R | R | Yes |
| VP-SV-17 | Shrimp hepatopancreas | *V. parahaemolyticus* | - | - | - | + | I | S | No |
| VM-SV-18 | Shrimp hepatopancreas | *V. mimicus* | + | - | - | - | I | I | Partial |
| VV-SV-19 | Shrimp hepatopancreas | *V. vulnificus* | - | + | - | - | S | I | No |
| VF-SM-20 | Shrimp muscle | *V. fluvialis* | + | - | - | - | I | S | No |
| VF-SM-21 | Shrimp muscle | *V. fluvialis* | - | - | + | - | S | S | No |
| VF-SM-22 | Shrimp muscle | *V. fluvialis* | + | - | - | - | R | I | Partial |
| VA-SM-23 | Shrimp muscle | *V. alginolyticus* | - | - | - | + | S | I | No |
| VA-SM-24 | Shrimp muscle | *V. alginolyticus* | + | - | - | - | I | I | Partial |
| VP-SM-25 | Shrimp muscle | *V. parahaemolyticus* | - | + | - | - | S | S | No |
| VF-W-26 | Water | *V. fluvialis* | + | - | - | - | R | R | Yes |
| VF-W-27 | Water | *V. fluvialis* | - | + | + | - | S | I | No |
| VA-W-28 | Water | *V. alginolyticus* | + | - | - | - | I | S | No |
| VA-W-29 | Water | *V. alginolyticus* | - | - | + | - | S | S | No |
| VV-W-30 | Water | *V. vulnificus* | + | - | - | - | S | I | No |
| VF-H-31 | Human stool | *V. fluvialis* | - | - | + | - | I | S | No |
| VF-H-32 | Human stool | *V. fluvialis* | + | - | - | - | I | I | Partial |
| VF-H-33 | Human stool | *V. fluvialis* | - | - | - | + | S | S | No |
| VA-H-34 | Human stool | *V. alginolyticus* | + | - | - | - | R | R | Yes |
| VA-H-35 | Human stool | *V. alginolyticus* | - | + | - | - | S | I | No |
| VD-H-36 | Human stool | *V. damsela* | + | - | - | - | I | S | No |
| VF-SV-37 | Shrimp hepatopancreas | *V. fluvialis* | - | - | - | + | S | S | No |
| VA-SV-38 | Shrimp hepatopancreas | *V. alginolyticus* | + | - | - | - | R | I | Partial |
| VF-W-39 | Water | *V. fluvialis* | - | + | - | - | I | I | Partial |
| VP-SV-40 | Shrimp hepatopancreas | *V. parahaemolyticus* | + | - | - | - | R | R | Yes |

**References:**

Centers for Disease Control and Prevention. (n.d.). *Cholera and Other Vibrio Illness Surveillance (COVIS)*. 2019. <https://www.cdc.gov/vibrio/php/surveillance/annual-summary-2019.html>

World Health Organization (2017) Chole ra outbreaks response field manual. https://www.who.int/publications/i/item/cholara-outreach-response-field-manual
